# Next-Generation Functional Nanoplatforms: Engineering Plant-Derived Extracellular Vesicles for Targeted Bioactive Delivery and Health Applications

**DOI:** 10.3390/molecules31203754

**Published:** 2026-10-19

**Authors:** Farhang Hameed Awlqadr, Mohammed N. Saeed, Ammar Badran Ramddan, Ahmad F. Hama Sdiq, Hamza Kamaran Hama, Md. Sakhawot Hossain, Sobia Noreen, Hassan Raza, Attila Péter Kiss

**Affiliations:** 1 Department of Food Biotechnology, Albert Kázmér Faculty of Agricultural and Food Sciences, Széchenyi István University, 9200 Mosonmagyaróvár, Hungary; kiss.attila.peter@sze.hu; 2 Department of Medical Laboratory Technique, Kifri Technical Institute, Garmian Polytechnic University, Kifri City, Sulaimaniyah 46001, Iraq; mohammed.noori@gpu.edu.iq; 3 Department of Food Science, College of Agriculture, University of Basrah, Basrah 61004, Iraq; ammar.ramddan@uobasrah.edu.iq; 4 Department of Medical Laboratory Science, College of Health Sciences, Lebanese French University, Erbil 44001, Iraq; ahmadfatih00624033@gmail.com; 5 Clinical Biochemistry, College of Science, University of Halabja, Halabja 46018, Iraq; hamza.hama@uoh.edu.iq; 6 Department of Nutrition and Food Engineering, Daffodil International University, Birulia, Dhaka 1216, Bangladesh; sakhawot.jnft@gmail.com; 7 University of Illinois Cancer Center, University of Illinois Chicago, Chicago, IL 60612, USA; sobianoreen07@gmail.com; 8 Faculty of Food Science and Nutrition, Bahauddin Zakariya University, Multan 60800, Pakistan; sh.raza40@yahoo.com

**Keywords:** plant-derived extracellular vesicles (PDEVs), bioactive delivery, functional foods, nanocarriers, cross-kingdom communication, precision nutrition, gut microbiota, nanobiotechnology

## Abstract

Plant-derived extracellular vesicles (PDEVs) are being recognized as a capable novel class of natural nanocarriers that have important uses in delivering bioactives and facilitating innovations in functional foods and precision health technologies. PDEVs are lipid-bilayer-enclosed vesicular structures that commonly occur at the nanoscale, although their reported sizes vary depending on the plant source, isolation procedure, and measurement method. They contain diverse biomolecules, including lipids, proteins, microRNAs, and phytochemicals, and have been investigated for their potential roles in interspecies communication and the delivery of bioactive compounds. Compared with some conventional nanocarriers, PDEVs have shown favorable biocompatibility and stability under certain experimental conditions; however, these properties are dependent on the plant source, isolation method, cargo, formulation, and experimental model; therefore, they are uniquely positioned for oral delivery systems. This review presents a complete picture of the process of PDEV biogenesis, the molecular architecture of PDEVs, and their cargo. We evaluate and compare the source materials of PDEVs to determine whether they will influence how effectively the bioactive agent is delivered to its intended target and produce the therapeutic effect. Additionally, we present a review of the advances in technology for the analysis of PDEVs: methods of extraction, purification, and characterization of PDEV; current and future techniques for loading cargo into PDEVs; methods of modifying the surfaces of PDEVs; and methods of developing, producing, and delivering hybrid systems of PDEVs. Finally, a comprehensive review of the use of PDEVs in delivering nutraceuticals, modulating the gut microbiome, delivering probiotic systems, and exhibiting anti-inflammatory, antioxidant, and anti-cancer effects. Even as these positive advancements occur, challenges related to scalability, standardization, and purity remain significant barriers to industrial translation. PDEVs are well-positioned to serve as a sustainable, next-generation nanoplatform at the intersection of nanotechnology, food science, and biomedicine. PDEVs also provide unprecedented opportunities to advance functional foods and precision nutrition.

## 1. Introduction

Extracellular vesicles (EVs) are small, lipid-based particles naturally released from living organisms, which are responsible for mediating communication between cells by transferring proteins, lipids, nucleic acids, and metabolites [1,2]. Plant-derived extracellular vesicles (PDEVs) are a new class of bioactive nanovesicles with distinctive compositions and functions originating from edible plants [3,4]. PDEVs typically occur within the nanoscale range, with reported sizes varying among plant sources, and consist of a lipid bilayer that encapsulates plant-based bioactive compounds, such as polyphenols, miRNAs, and secondary metabolites, thereby supporting the transfer of bioactive molecules across biological kingdoms [5]. Research on EVs has historically focused on mammalian exosomes; in the past, exosomes were considered by-products of cellular processes. However, early scientific research established that exosomes are active signaling entities with therapeutic capabilities [6]. As the scientific community began to understand these changes, interest has expanded into plant systems. The bioactivity and potential for scalability or compatibility with human use of plant nanovesicles are being studied [2]. By moving away from mammalian-derived vesicles and toward plant-based vesicles, the scientific community is facilitating a broader shift toward using safe, sustainable, and food-based nanocarriers.

Although nano-delivery technologies have made significant advancements, established delivery systems, such as liposomes, nanoemulsions, and polymeric nanoparticles, remain limited in many ways, hindering their use in nutraceutical and food-grade applications due to poor stability under GI tract conditions, potential toxicity, low targeting efficacy, and high production costs [7]. Therefore, these systems are limited; however, at the same time, PDEVs have demonstrated favorable biocompatibility and relatively low immunogenicity, while their reported stability under physiological and intestinal conditions supports their potential for use in oral delivery [8,9]. Recent work suggests that PDEVs are naturally occurring nano-delivery platforms that encapsulate and protect bioactive compounds, thereby improving their solubility, bioavailability, and targeted delivery. For example, plant-derived extracellular vesicles have been shown to improve both the stability and bioaccessibility of hydrophobic nutraceuticals, such as curcumin, and enable targeted cellular uptake [10]. Several studies have also shown that PDEVs derived from various fruits and leafy vegetables provide multiple bioactivities, including antioxidant, anti-inflammatory, and metabolic activities [11,12]. This review will provide an integrative overview of the role of PDEVs in functional food systems and health-related applications. By integrating concepts from food science and nanotechnology, this review highlights the potential of PDEVs to serve as both carriers of bioactive compounds and bioactive materials. Therefore, PDEVs may provide promising opportunities for precision nutrition and advancing sustainable nutraceutical innovation. The evidence discussed in this review is primarily focused on plant-derived extracellular vesicles and plant-derived vesicle-like nanoparticles. Vesicular systems originating from fungi, algae, bacteria, or mammals, as well as engineered hybrid systems, are considered distinct from PDEVs because of their different biological origins and compositions. Such studies are included only where they provide relevant comparative or contextual information and are not considered direct evidence for PDEV-specific properties or biological effects.

### Literature Search and Selection

A systematic literature search was conducted in PubMed, Scopus, Embase, and the Web of Science Core Collection from database inception to 1 April 2026. Controlled vocabulary, where available, was combined with database-specific title, abstract, and keyword terms related to plant-derived extracellular vesicles, plant extracellular vesicles, plant-derived exosomes, exosome-like nanoparticles, nanovesicles, isolation, characterization, molecular cargo, gastrointestinal fate, engineering, cargo loading, drug delivery, functional foods, gut microbiota, and therapeutic applications. English-language publications were selected. Complete database-specific search strategies, search dates, platforms, and record counts are provided in Supplementary Table S1.

All retrieved records were exported to EndNote and merged into a single reference library. EndNote was used to identify and remove duplicate records on the basis of DOI, PMID, database accession number, and title. Potential near-duplicates with minor differences in titles or bibliographic details were checked manually against the authors, publication year, journal, volume, issue, and page range. Of the 6711 records identified, 3198 duplicates were removed before screening, leaving 3513 unique records for title and abstract screening.

Study selection was performed in two stages. Titles and abstracts were first assessed for relevance to the scope of the review, resulting in the exclusion of 3154 records. Full texts were sought for 359 reports; 89 could not be retrieved, and 270 reports were assessed for eligibility. At the full-text stage, 127 reports were excluded because of insufficient methodological information (*n* = 30), the absence of a comparable characterization or relevant outcome (*n* = 61), or the absence of clear food, nutraceutical, or health relevance (*n* = 36). A total of 143 studies were included in the comparative synthesis.

**Figure 1:**
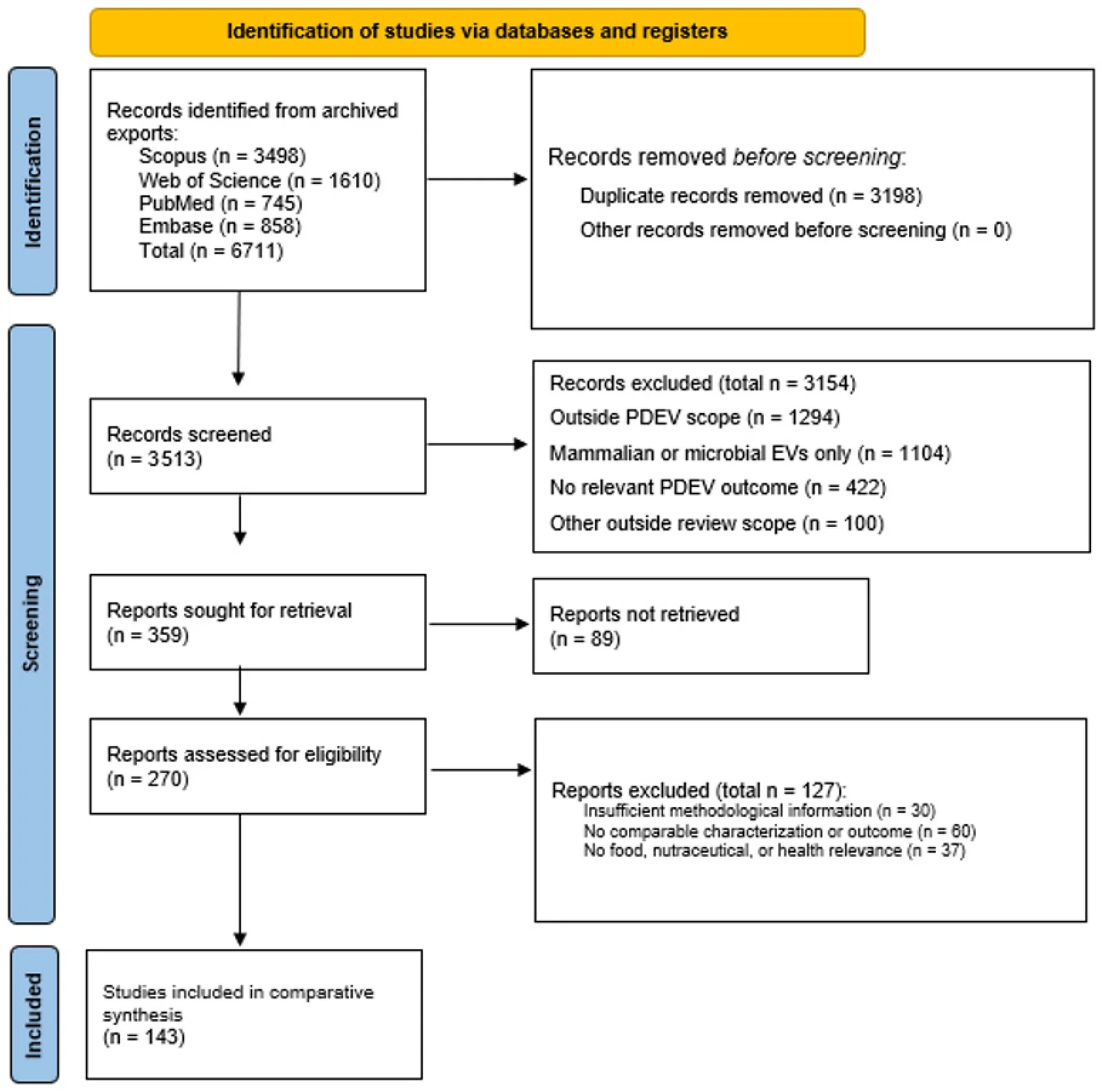
PRISMA 2020 flow diagram of study identification, screening, eligibility assessment, and inclusion [13].

Original studies were eligible when they reported experimental evidence on PDEV biogenesis, isolation or purification, physicochemical characterization, molecular composition, gastrointestinal behavior, engineering, delivery performance, or biological activity. Review articles were used for background information and citation tracking but were not treated as primary evidence in the comparative synthesis. Mechanically generated plant nanoparticles and non-plant extracellular vesicles were excluded from the PDEV evidence base unless they were explicitly discussed as comparative systems. The selection process is summarized in Figure 1.

## 2. Biogenesis and Molecular Architecture of PDEVs

### 2.1. Formation Pathways

Plant-derived extracellular vesicles (PDEVs) are heterogeneous membrane-bound particles that may arise through different cellular pathways. Evidence for the classical multivesicular body (MVB)-associated pathway and unconventional secretion pathways has been reported in plants; however, the specific biogenesis pathway cannot generally be assigned to an isolated PDEV preparation solely on the basis of its size, morphology, or isolation method. In the classical pathway, plant cells form MVBs through the endosomal system, in which inward invagination of the endosomal membrane generates intraluminal vesicles that can subsequently be released as extracellular vesicles [14]. Evidence supporting MVB-associated secretion includes the detection of lipid and protein features associated with endosomal pathways in characterized plant extracellular vesicle populations [15].

**Figure 2:**
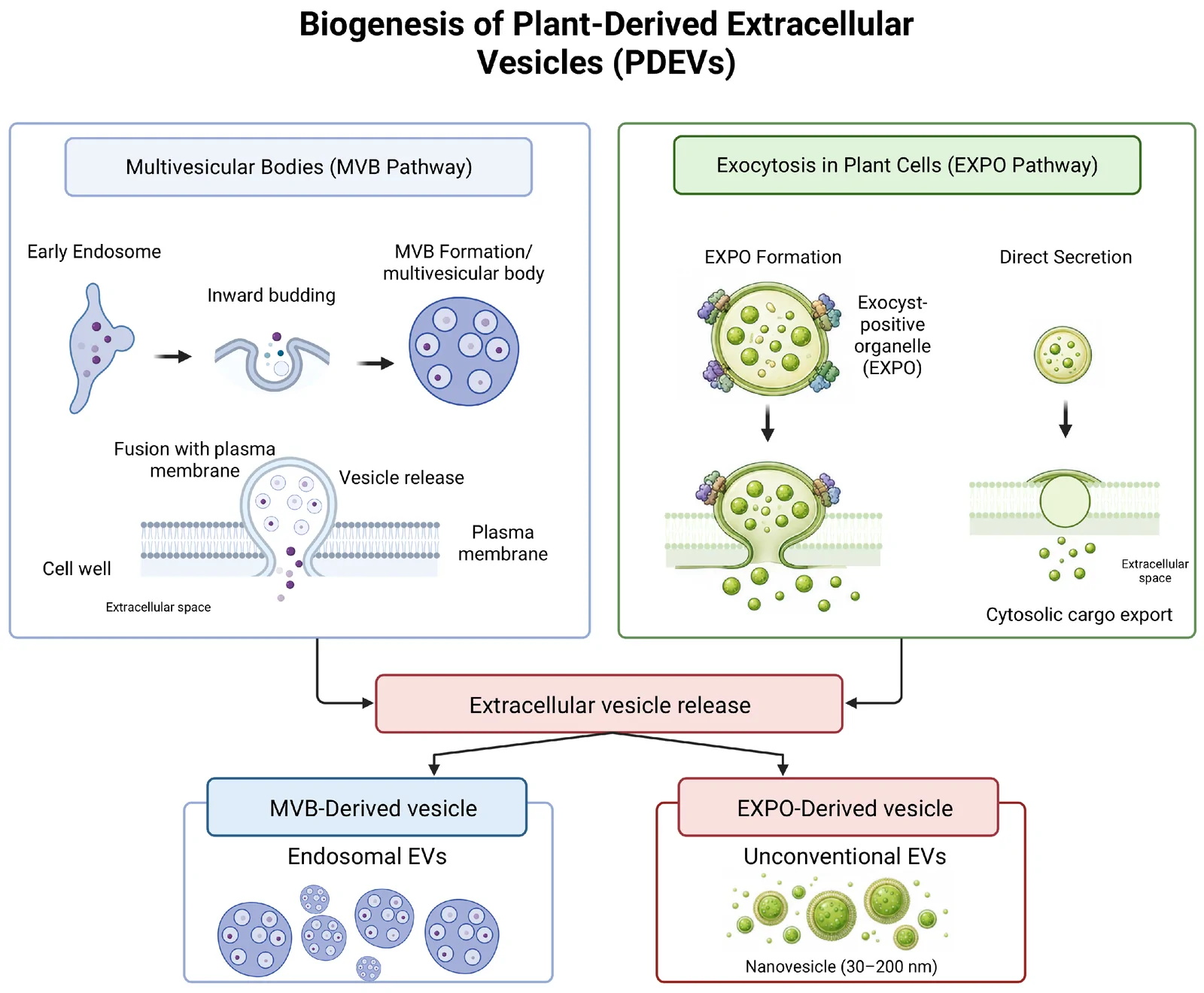
Biogenesis pathways of plant-derived extracellular vesicles (PDEVs). Blue/purple and green indicate the MVB and EXPO pathways, respectively; arrows indicate pathway progression and vesicle release.; created in BioRender. Awlqadr, F. (2026) https://BioRender.com/w3resjw.

MVB-mediated cellular secretion is important for intercellular communication and plant defense. MVBs provide a mechanism through which PDEVs can participate in intercellular signaling and responses to environmental stress and pathogen invasion. Therefore, PDEVs may play important roles in biological processes beyond the transport of molecular cargo [16]. Furthermore, employing density gradient separation has enabled the identification of several distinct vesicle populations, with several corresponding to those derived from multiple vesicle bodies (MVBs), thereby demonstrating that endosomal pathways are involved in their formation. In addition to forming through the classical MVB pathway, PDEVs are formed through exocytosis via otherunconventional secretion pathways. An example of this is the EXPO pathway, which transports cytoplasmic components to the extracellular space without the involvement of the Golgi apparatus via exocyst-positive organelles [17]. The EXPO pathway is an adaptation of plants that adds to the diversity of vesicles and their capabilities. In addition, vesicle formation in plants can depend on cellular metabolic status, developmental phase, and environmental conditions. For instance, the rate of vesicle secretion and the components present in the vesicles are both influenced by active growth and by periods of stress, demonstrating dynamic regulation of biogenesis [18]. Another advantage of using plant cell suspension culture systems is that they have been shown to produce more vesicles, thereby providing a controlled environment for studying vesicle biogenesis and improving scalability across diverse applications [19]. A major difference, however, between PDEVs and mammalian extracellular vesicles is their plant origin; consequently, they possess unique mechanisms of biosynthesis and cargo selection that facilitate cross-kingdom signaling and enhance vesicle stability [20]. Overall, PDEV biogenesis involves both classical endosomal and unconventional secretory pathways, which may contribute to the diversity of vesicle populations, cargo composition, and biological functions. These routes contribute to vesicle diversity and may influence cargo composition and biological function Figure 2.

### 2.2. Structural Characteristics and Molecular Cargo of Plant-Derived Extracellular Vesicles (PDEVs)

**Figure 3:**
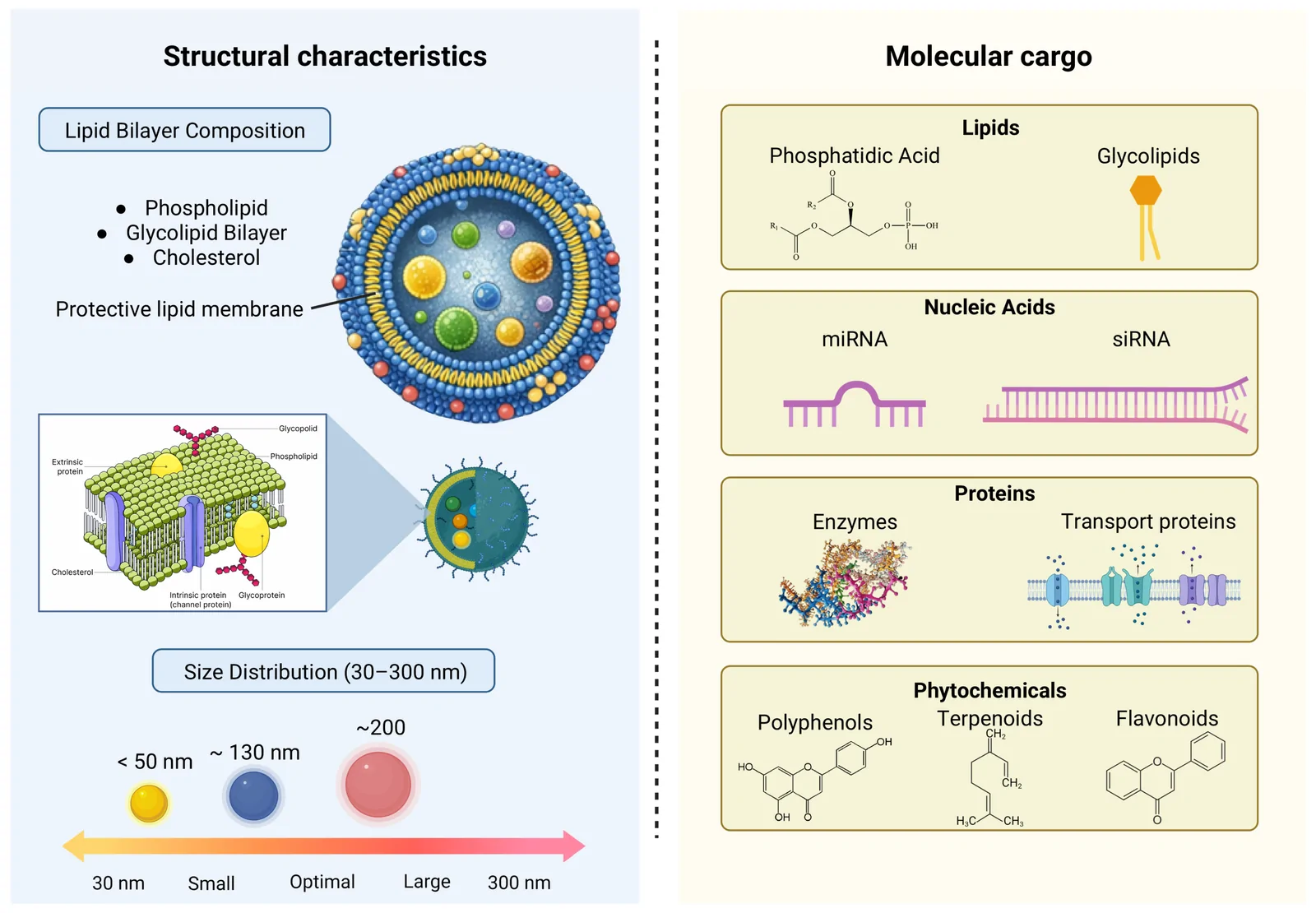
Structural characteristics and molecular cargo of plant-derived extracellular vesicles (PDEVs). Blue and yellow panels show structural characteristics and molecular cargo, respectively. Colored spheres illustrate vesicle sizes, and the gradient arrow indicates increasing diameter. Molecular symbols schematically represent the indicated cargo classes. Created in BioRender. Awlqadr, F. (2026) https://BioRender.com/kmmxqyg.

The extracellular vesicles derived from plants have unique structural and molecular properties that make them suitable for use as naturally occurring nanocarriers. They have a simple structure, with a lipid bilayer that serves as both the structural boundary between the vesicle interior and the outside. Various types of lipids, such as phospholipids, glycolipids, and sterol-like lipids, are present in the membrane. The varied lipid composition is responsible for membrane organization and, therefore, for interactions between PDEVs and recipient cells. PDEVs share structural similarities with mammalian EVs but have a distinct molecular composition due to their plant origin, which confers distinct biological properties [20]. Lipidomic analysis has revealed the presence of major lipid components of PDEVs, such as phosphatidic acid, phosphatidylcholine, ceramides, and glycolipids [21]. These lipids are important for membrane organization and fluidity and may affect vesicle-cell interactions and cellular uptake. PDEVs also differ in physical properties depending on the source plant and isolation conditions. Many reported preparations fall within the nanoscale, with numerous studies describing particles in the approximate range of 30–300 nm; however, substantial variation has been reported among different preparations. Therefore, particle size alone should not be considered a definitive criterion for assigning PDEV identity, particularly when larger plant-derived nanovesicles or nanoparticles are reported [22]. Smaller vesicles can show good cell internalization properties, while larger vesicles can offer a higher internal volume for molecular cargo. For example, plant-derived nanovesicles from walnut and onion have been reported with average sizes of ~130–140 nm and ~195 nm, respectively [23]. In addition to structural properties, PDEVs can carry an abundance of molecular cargo, which may be responsible for their biological activity. PDEVs are rich in various lipids, proteins, nucleic acids, and phytonutrients that could be responsible for their biological action. Other lipids that bind to PDEVs may also be involved in cellular signaling and may affect inflammatory, metabolic, and intercellular communication pathways [24]. Besides membrane structure, PDEVs are filled with a variety of molecular cargo, including lipids, proteins, nucleic acids, and plant phytochemicals. This cargo is contained in or linked to the vesicular architecture and is a major source of molecular diversity among PDEVs. Proteomic studies have shown that these vesicles contain enzymes, transport proteins, stress-response proteins, and metabolic proteins, indicating their biological origin. Nucleic acids, such as microRNAs (miRNAs) and small interfering RNAs (siRNAs), have also been found in PDEVs. PDEVs have been shown to have a variety of small RNA species, as suggested by sequencing studies, and therefore contain a complex molecular cargo [25]. PDEVs can also be enriched with plant-specific phytochemicals and plant metabolites such as polyphenols, terpenoids, and flavonoids. The composition and concentration of these compounds may vary with plant source and may contribute to the functional diversity of PDEVs. All these properties distinguish PDEVs from traditional synthetic nanovectors used for drug delivery. One should be able to distinguish biologically secreted PDEVs from other plant-derived, nanoscale materials produced during mechanical disruption or extraction. Mechanical processing of plant tissues can yield heterogeneous nanoparticles or vesicular structures that may be present in the isolated fraction alongside natural extracellular vesicles secreted by the tissues [26]. Thus, the mere presence of nanoscale particles is not enough to define their biological origin as EVs. In this review, the term PDEV is primarily used for plant-derived extracellular vesicles that have been reported or characterized as vesicular structures of extracellular origin. Preparations generated through mechanical disruption, blending, homogenization, or extraction are described according to the terminology used in the original studies, such as plant-derived nanovesicles or nanoparticles, when their extracellular origin has not been established. This distinction is important because mechanical processing can generate heterogeneous membrane-derived particles that cannot be assumed to represent secreted extracellular vesicles. The main structural features and molecular contents of PDEVs are summarized in Figure 3.

Terminology should also reflect the level of evidence available for vesicle identity and biogenesis. Following current extracellular-vesicle reporting guidance, the generic term “extracellular vesicle” (EV) is preferable when the specific biogenesis pathway has not been demonstrated. Terms such as “exosome” should therefore be reserved for preparations for which an endosomal/MVB origin is supported experimentally, whereas “exosome-like nanoparticle,” “nanovesicle,” or “plant-derived nanoparticle” should not be treated as synonymous with bona fide EVs. This distinction is particularly important for plant-derived preparations obtained by blending, homogenization, pressing, or other forms of tissue disruption, because such procedures can release intracellular membranes and generate heterogeneous vesicular and non-vesicular particles. Accordingly, preparations obtained through tissue disruption are described in this review according to the terminology and evidence provided by the original studies, and their biological origin is not assumed to be equivalent to naturally secreted extracellular vesicles. These distinctions are consistent with the principles of MISEV2023, which recommend cautious use of biogenesis-related terminology and emphasize the need to distinguish EVs from co-isolated non-vesicular extracellular particles [27].

## 3. Sources and Comparative Profiling of PDEVs

### 3.1. Edible Plants (Fruits and Vegetables)

Edible fruits and vegetables are among the most readily available sources of PDEVs, especially in the context of functional foods and oral delivery. Grapes, citrus fruits, apples, bananas, strawberries, blueberries, garlic, broccoli, and various other edible plants have been studied as the sources of vesicles containing different molecules and biological activity [28]. The greatest benefit of their use is the availability of lots of edible biomass and the fact that the source material has been eaten by humans in the past. The suitability of an edible plant as a PDEV source, however, cannot be measured just by the abundance or reported biological activity of the plant—such as vesicle yield, size distribution, and molecular composition—and functional properties vary greatly depending on the species and preparation method. Grape vesicles have been relatively well studied among edible sources, with a range of reported properties, including nanoscale dimensions, phenolic cargo, anti-inflammatory activity, stability, and effects on host–microbiome interactions. Vesicles derived from citrus species also have a somewhat wide set of explored functions. Lemon and orange vesicles have been linked to “macrophage regulation”, “wound healing,” “antioxidant responses,” and “modulation of intestinal microbial activity” [29]. Investigations of grape and citrus sources were repeated across various biological models, providing firmer grounds for their evaluation as potential sources than isolated reports from less extensively studied plant sources. However, it should be noted that direct comparisons are not always straightforward, as various studies use different methods for isolation, vesicle characterization, models, and doses. Variation in biological activity from edible plant sources may be due more to differences in cargo in vesicles than particle size. Vesicles extracted from grapefruit, for instance, have been compared to those from tomato in terms of protein-loading capacity and efficiency of protein delivery, and vesicles derived from grapefruit have been found to be more protein-loading [30]. Likewise, broccoli vesicles are rich in lipids, rich in sulforaphane, and grape and pomegranate vesicles are rich in phenols and anthocyanins associated with lipids [31]. The differences found may provide a molecular basis for the distinct biological responses reported across plant sources. Still, the activity of individual cargo molecules and their contribution to the observed activity have not always been experimentally distinguished from the effects of the vesicular structure itself. The data summarized in Table 1 summarizes significant differences in particle size (ranging from vesicles smaller than 50 nm to approximately 400 nm). Caution should be used when interpreting this variation, as it depends on the biological heterogeneity of the vesicle preparation and the method of preparation and measurement of vesicles. Therefore, the reported size of larger or smaller should not be taken as evidence of better biological performance per se. For instance, blueberry vesicles have been reported to be stable and active in the intestine, despite the fact that vesicles are reported to have a relatively wide size distribution, while other sources with more narrow size distributions have been mainly used for cargo delivery [32]. Overall, edible plants offer an appealing and promising biomass platform, but there is currently insufficient evidence to support the selection of a single optimal PDEV source. Grape and citrus sources are well supported by experiments to date, while a few other edible sources are supported primarily by individual studies. Comparative studies with the same isolation, characterization, dosage, and biological testing procedures are required in the future to determine whether differences in PDEV activity are due to plant source, molecular cargo, vesicle heterogeneity, or experimental methodology.

### 3.2. Medicinal and Functional Plants

Another valuable source of PDEVs is medicinal and functional plants, whose vesicles can be rich in phytochemicals and other molecular components that reflect the plant’s biological properties. Ginger (*Zingiber officinale*), ginseng (*Panax ginseng*), turmeric (*Curcuma longa*), aloe (*Aloe* spp.), saffron (*Crocus sativus*), and fenugreek (*Trigonella foenum-graecum*) have been investigated for their potential therapeutic and delivery-related applications. The amount of evidence, however, varies considerably across sources, and reported effects on biological systems should be considered in the context of each preparation’s degree of characterization and experimental validation. Ginger vesicles are among the most well-studied medicinal-plant PDEVs. They have been reported to have anti-inflammatory cytokine inhibitory activity, immune modulating activity, fast cellular uptake, and therapeutic cargo delivery properties [42]. They are relatively uniform at the nanoscale, and they have been studied in multiple models of inflammation, cancer, and drug delivery, offering comparatively strong evidence of their versatility. However, the results of these studies cannot be directly compared quantitatively due to the different types of vesicles used and the varying experimental conditions. Another well-studied type of vesicle is the ginseng-derived vesicle, which is known to contain bioactive molecules such as ginsenosides. Anti-inflammatory, immunomodulatory, anti-cancer, and blood–brain barrier-related applications have been reported in studies [43,44]. The finding of many protein-containing vesicles suggests high molecular complexity in ginseng. It is difficult to know which individual components are responsible for specific biological effects; however, because of the presence of many proteins and phytochemicals. Therefore, the therapeutic effect of ginseng vesicles cannot be said to be due to any one component. Turmeric vesicles have attracted considerable interest due to their curcuminoid content and purported anti-cancer and metabolic actions [45,46]. Vesicles derived from aloe have also been explored for their antioxidant, photoprotective, and tissue-repair properties [47]. The biological outcomes of these preparations vary, and comparisons between studies are difficult due to variations in plant species, vesicle isolation, cargo composition, and experimental models. The broad particle-size distribution reported for some aloe-based preparations also suggests potential differences in biological behavior among particle sets. Other medicinal plants also show such a relationship between plant-specific cargo and PDEV activity, for example, saffron and fenugreek. Saffron-derived vesicles have been shown to contain anthocyanins and miRNAs, which are related to immune response modulation [48] and fenugreek-derived vesicles have been explored as drug carriers for antibiotics and have exhibited antibacterial, anti-inflammatory, and antifibrotic properties [49]. It is demonstrated here that these intrinsic vesicular properties can be combined with the external loading of therapeutic compounds. The evidence cited for these sources, however, is still less than that available for ginger, ginseng, or turmeric. Table 2 shows the variations among medicinal-plant PDEVs in their molecular contents, particle sizes, and biological activities. These differences might be caused by differences in plant metabolism, cargo sorting, vesicle heterogeneity, or analytical methods, as well as by differences in tissue type and in the isolation protocol used [50]. Therefore, any biological activity observed with one preparation of plant vesicles does not necessarily apply to all preparations from the same plant species. Medicinal and functional plants are potential sources for PDEV implementation, but data are not consistent across plant species. Ginger and ginseng are relatively well supported in experimental studies, while other sources are supported by fewer studies or by specific disease models [51]. One key question that remains unanswered is the absence of a standardized comparative study to assess various plant sources under the same isolation, characterization, dosing, and biological conditions. These studies would also help identify authentic source-specific biogenic variations from methodological variation and create a better correlation among plant origin, molecular cargo, vesicle characteristics, and biological activity.

**Figure 4:**
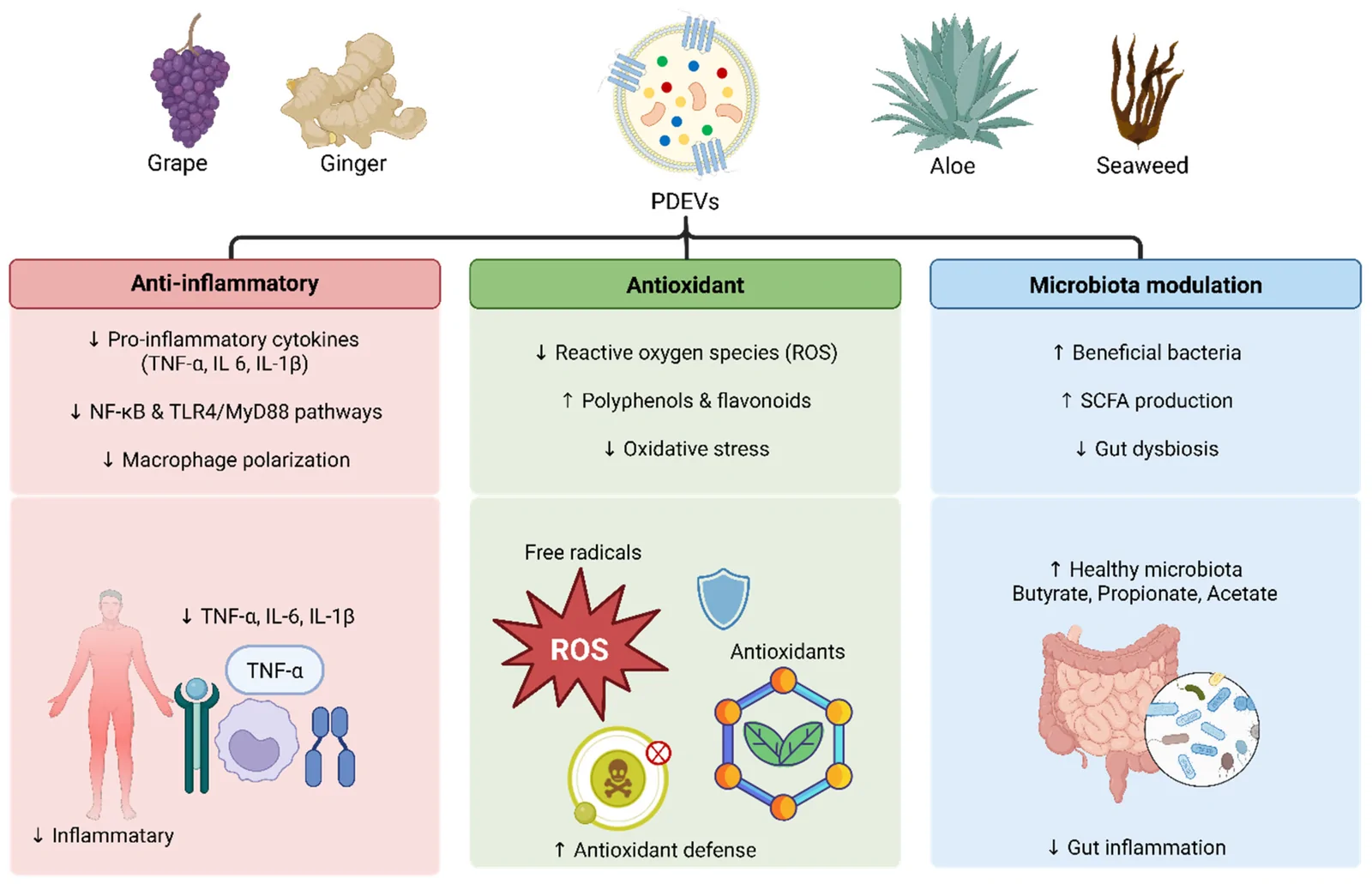
Comparative functional properties of plant-derived extracellular vesicles (PDEVs). ↑ and ↓ indicate increases and decreases, respectively. Pink, green, and blue panels represent anti-inflammatory effects, antioxidant effects, and microbiota modulation, respectively. Icons depict plant sources, vesicles, and biological targets; the shield and crossed-out symbol indicate protection and inhibition, respectively. Awlqadr, F. (2026) https://BioRender.com/yv35hfl.

PDEVs possess a variety of intrinsic functional activities, such as anti-inflammatory, anti-oxidative, and microbiota-modulatory activities. The properties depend on the molecular makeup of the vesicles and their plant source. Therefore, the lipid, protein, nucleic acid, and phytochemical loads carried may account for differences in the functional profiles of PDEV preparations. PDEVs from various medicinal plants, such as grape, ginger, aloe, etc., have been found to possess anti-inflammatory activity. These vesicles have been linked to the suppression of pro-inflammatory cytokines, including IL-1β, IL-6, and TNF-α, and to modulation of inflammatory signaling pathways, like NF-κB and TLR4/MyD88 [45,46]. Grape-based vesicles (with high phenolic content) have been shown to have anti-inflammatory activity in oral tissues. Likewise, there is a report on the suppression of IL-6 expression by vesicles derived from ginger and the effect of aloe-derived vesicles on cytokine secretion and macrophage polarization [40]. PDEVs also show antioxidant properties because of the presence of polyphenols, flavonoids, and other metabolites with redox properties. Exposure to vesicles derived from plants has been shown to decrease intracellular reactive oxygen species (ROS) and oxidative damage in several studies [57]. Another key intrinsic characteristic of the PDEVs is their ability to modify the gut microbiota. Intestinal microorganisms can interact with other molecular components, such as vesicular lipids, miRNAs, and metabolites, and affect microbial composition and metabolism. PDEVs have been reported to promote the proliferation of beneficial bacterial populations and increase the production of short-chain fatty acids (SCFAs), which are known to positively affect intestinal physiology. As a result, the interactions of PDEVs do not just include mammalian cells but also the intestinal microbial ecosystem [58]. Figure 4 summarizes the major intrinsic functional properties of PDEVs, including anti-inflammatory, antioxidant, and microbiota-modulating activities.

## 4. Isolation, Purification, and Standardization Strategies

### 4.1. Conventional Methods

#### 4.1.1. Differential Ultracentrifugation

**Figure 5:**
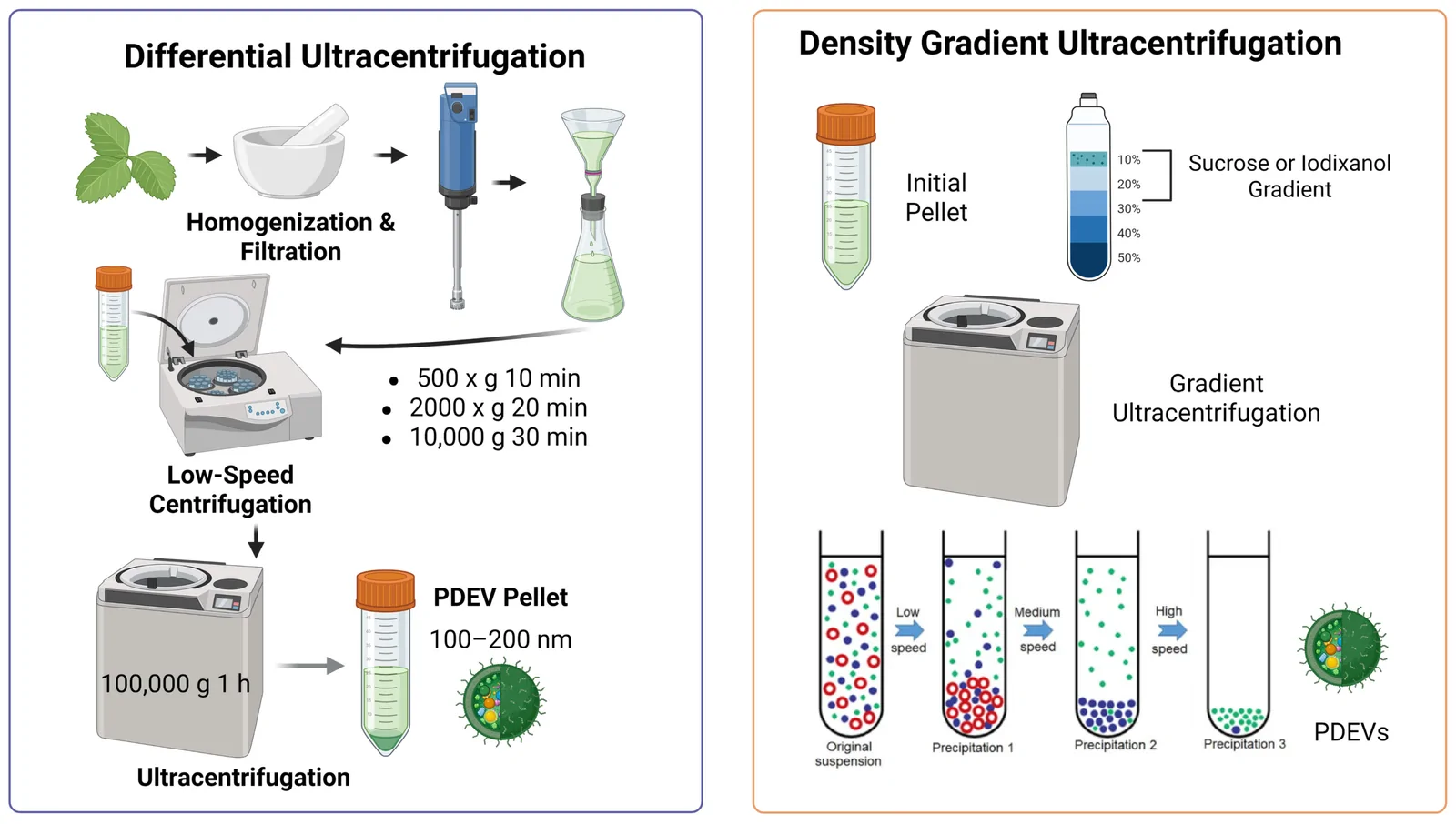
Comparative guide to plant-derived extracellular vesicle (PDEV) isolation. Left: plant tissue grinding, homogenization, filtration, centrifugation, and ultracentrifugation (100,000× *g*, 1 h) to recover vesicles. Right: density-gradient separation and sequential centrifugation at increasing speeds. Arrows indicate processing steps; colored particles represent different suspension components, blue shading indicates gradient concentrations, and green vesicle icons depict recovered PDEVs. Purple and orange borders distinguish the two panels. Created in BioRender. Awlqadr, F. (2026) https://BioRender.com/86vy1ch.

One of the most common methods for isolating PDEVs from fruits, medicinal plants, juices, and plant tissues is differential ultracentrifugation (DUC). The technique includes successive centrifugation steps to eliminate vesicles, bigger organelles, and cellular debris, as well as isolating intact cells, which are then pelleted at high centrifugal forces [59,60]. Generally, the plant material or juice is homogenized in a cold isotonic buffer, centrifuged at low speed, and then ultracentrifuged at approximately 100,000–150,000× *g* to isolate the vesicular fraction. There are several practical benefits of DUC for PDEV isolation on the lab scale. It can be used with almost any plant material and does not require special chromatographic materials, making it relatively available in laboratories equipped with an ultracentrifuge. The method can be used to recover enough vesicles for further physicochemical or biochemical study. For instance, vesicles isolated from ginger exhibited significant recovery, and vesicles isolated from orange maintained their biological activity and morphology after isolation [61]. Recovery of structurally complex tissues, such as roots, can be improved by additional processing, such as enzyme pretreatment or multiple ultra-centrifugations. Although these benefits exist, DUC cannot fully separate PDEVs from similarly settling contaminants. Protein aggregates, polysaccharides, cellular components, and other plant-based nanoparticles can be found in the vesicular fraction, especially if complex plant matrices are employed [62,63]. Thus, the apparent biological activity of the DUC preparation may be the combined result of vesicle formation and the co-isolation of molecular components. This is especially relevant for mechanistic studies, where a specific biological effect must be attributed to the cargo associated with PDEV. Therefore, depending on the intended use, the suitability of DUC must be determined. DUC is a practical and reasonably powerful isolation method for exploratory studies and applications where moderate purity is acceptable. Some preparations intended for detailed molecular characterization, for studying mechanisms, or for use in applications requiring higher purity might benefit from an additional purification step, such as density gradient ultracentrifugation or column chromatography on Sephadex. DUC also requires a certain amount of time for processing and specialized ultracentrifugation equipment, which can restrict its scalability for large volume production [59]. To sum up, DUC remains a valuable primary isolation technique due to its versatility and its ability to obtain fractions enriched in PDEV. However, the recovery, purity, processing time, and scalability indicate that DUC may not be optimal for all PDEV applications. The location of DUC relative to other PDEV isolation/purification approaches is shown in Figure 5. PDEV preparations can be further purified by density-gradient ultracentrifugation (DGUC) following differential ultracentrifugation. The method is based on the buoyant density of vesicles, and it is especially useful for complex matrices of plant origin consisting of proteins, polysaccharides, cellular components, and other plant-derived nanoparticles [64]. In a conventional method, a crude PDEV pellet collected from DUC is placed on a discontinuous sucrose or iodixanol gradient and ultracentrifuged for an extended period. Vesicles are separated from contaminants of different densities by migrating to the fractions according to their characteristic density (typically 1.13–1.19 g/mL) [65].

#### 4.1.2. Density-Gradient Ultracentrifugation

DGUC can offer a better degree of purification and more uniform physicochemical properties than DUC alone. For instance, vesicles purified using a sucrose-gradient approach yielded a highly purified population of vesicles with consistent physicochemical properties from ginseng [65]. Likewise, the lotus-derived vesicles prepared by DGUC had high purity and a relatively uniform size ranging from 130 to 160 nm as compared to the vesicles prepared by using only DUC [66]. This analysis justifies using DGUC when greater control over sample composition and vesicle characteristics is desired in downstream studies. The main benefit of DGUC is thus the reduction in contamination that can remain after the normal centrifugation procedure. This is especially significant in mechanistic studies and molecular characterization, where co-isolated proteins, polysaccharides, or other nanoparticles may interfere with the interpretation of the biological effects associated with vesicles. This enhanced purity could also prove useful for assessing the correlation of PDEV cargo with biological activity. DGUC is not always able to separate all plant-derived contaminants; however, additional purification steps can lead to sample loss and necessitate further recovery of the vesicular fractions. One of the drawbacks of DGUC is its relatively long processing time and higher operational complexity. This method is more labor-intensive than DUC alone due to gradient preparation, prolonged high-speed centrifugation, fraction collection, and vesicle recovery. Although it has the advantage of purity, it is not suitable for high-throughput or large-scale production due to its properties. For this reason, the use of DGUC is particularly well-suited to laboratory-scale preparations that usually require relatively high purity, whereas methods such as ultrafiltration and tangential-flow filtration would be better for larger-volume preparations. In summary, DGUC is a valuable compromise between purity and vesicle recovery, providing significant purification benefits over DUC alone but requiring more time and processing steps and offering less scalability. Therefore, its use with DUC is beneficial when relatively high-purity, reproducible preparations of PDEV are needed for biological studies or detailed characterization [60].

### 4.2. Advanced Techniques

#### 4.2.1. Size-Exclusion Chromatography (SEC)

For the purification of PDEVs based on their hydrodynamic size, a relatively gentle method, such as size-exclusion chromatography (SEC), can be used. The separation can be performed on a porous stationary phase, such as Sepharose CL-2B or Sepharose CL-4B, resulting in the elution of larger vesicular particles ahead of smaller proteins, metabolites, and other soluble components [67]. Generally, all plant extracts (such as fruit juice, plant decoction, and media from plant cell culture) are clarified and filtered prior to loading onto an equilibrated SEC column. Particles with vesicles can then be detected using particle- or protein-based methods such as nanoparticle tracking analysis (NTA) or protein assays. Mild SEC processing conditions are an important benefit, as they can help maintain the sensitivity of the molecular cargo and the structure of vesicles. SEC has been employed for turmeric-derived exosome-like nanoparticles and for PDEVs obtained from functional-food sources such as citrus and grapes. SEC has the advantage of minimizing the co-isolation of low-molecular-weight and soluble proteins; it can be used when preserving bioactive components, such as miRNAs and phytochemicals, is relevant, compared with the ultracentrifugation technique [67]. Yet there are significant drawbacks to the SEC. The separation relies mostly on particle size; that is, particles with hydrodynamic diameters comparable to PDEVs may not be effectively separated. Moreover, vesicles are dispersed throughout the eluted fractions, and the final product may be rather diluted and require further concentration steps, usually by ultrafiltration. Thus, while SEC may offer better sample quality, it comes at the expense of recovery and downstream processing. Therefore, the suitability of the SEC depends on the intended application. It is well-suited for studies where the molecular cargo is sensitive or for functional characterization but may not be preferred for studies that require large amounts of PDEVs due to lower recovery and dilution. SEC can then be used after a first concentration step (e.g., DUC or ultrafiltration) and not as a universal alternative to centrifugation-based isolation. In general, SEC is a valuable method for gentle processing and high purity, but due to the size-separation process, it is also limited for some complex plant matrices and larger-scale applications because of its relatively low recovery limit. It is most useful when there is a need to preserve the integrity of the vesicles, protect the contents from damage, and maintain yield [68].

#### 4.2.2. Ultrafiltration

Membrane ultrafiltration (UF) is a quick method for concentrating and partially purifying PDEVs using membranes with a molecular weight cut-off (MWCO) of 50–300 kDa. This process separates vesicles from smaller molecules based on size, using pressure or centrifugation [59]. Orange juice, ginger preparations, and plant cell culture media are among the plant extracts that can be pre-cleared using ultrafiltration units with polyethersulfone membranes. To make it more suitable for larger volumes, tangential flow filtration (TFF) is used, in which membrane fouling is minimized, and continuous filtration is possible. Overall, UF has been shown to offer benefits in processing speed, concentration, and scalability compared with ultracentrifugation. For instance, TFF-derived vesicles were extracted from oranges and exhibited similar physicochemical properties to those extracted by ultracentrifugation, but with improved processing capabilities and scalability [30]. UF is thus an appealing option for higher production of PDEVs from edible plants and bioreactor systems. Furthermore, the same strategy can be used to enable buffer exchange and concentration, thereby reducing the number of downstream processing steps. However, the purification is only partially achieved by UF because separation is largely based on molecular size. This means that contaminants with dimensions like PDEVs can remain in the preparation. Moreover, pressure and shear forces generated during membrane processing may damage or deform the vesicles, especially when processing conditions are not optimized. Another potential impact of membrane fouling may occur on the filtration performance, but TFF systems can minimize this effect. The appropriateness of UF, therefore, is influenced by processing efficiency, recovery, purity, and scalability. This is beneficial, especially for high-throughput or high-speed concentration, and an SEC or density gradient centrifugation purification step may be needed if high sample purity is required. Therefore, UF is better seen as a concentration/purification strategy that can be scaled up, but is not the universal solution to replace higher resolution purification techniques [69]. In summary, UF offers an essential advantage in processing PDEV production due to its rapid concentration, buffer exchange, and scalability. Nevertheless, the limitations of selective purification and possible effects of shear associated with the membrane should be taken into consideration when designing workflows for detailed characterization or therapeutic applications [69].

#### 4.2.3. Microfluidics-Based Isolation

**Figure 6:**
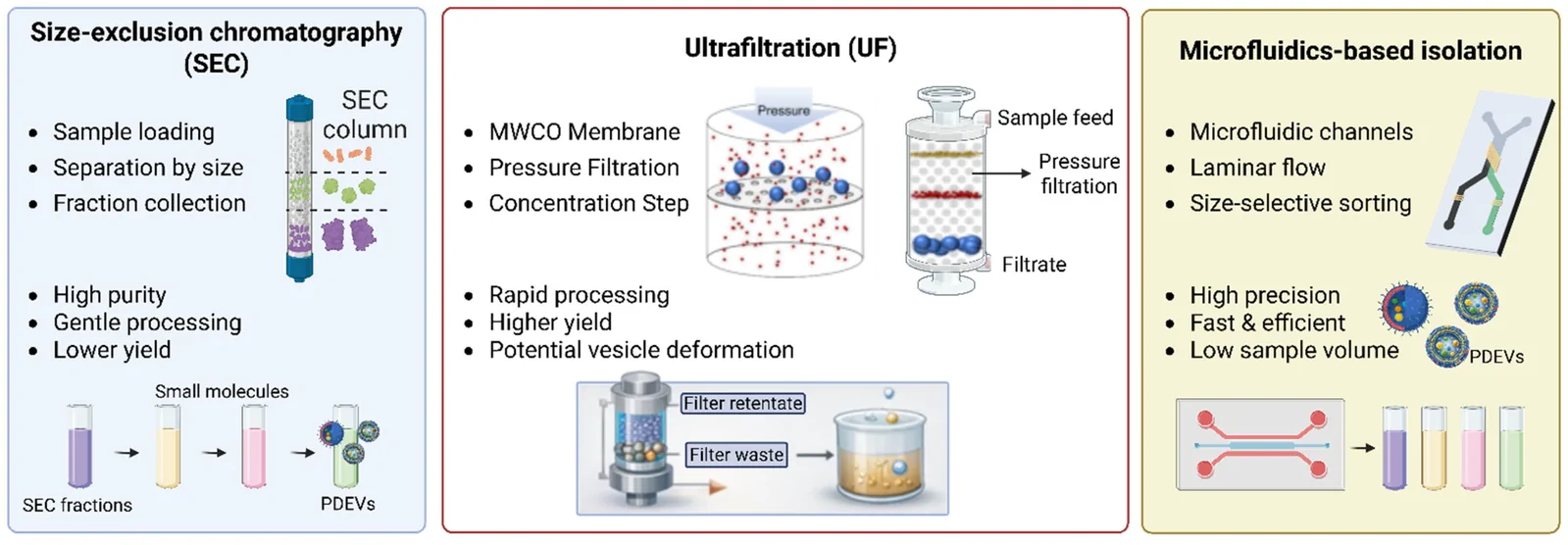
Advanced techniques for plant-derived extracellular vesicle (PDEV) isolation: size-exclusion chromatography, membrane filtration, and microfluidics. Arrows indicate flow and processing steps. Colored particles in the SEC column represent components of different sizes; colored collection tubes denote separate fractions, and vesicle icons represent PDEVs. Panel colors distinguish the techniques, created in BioRender. Awlqadr, F. (2026) https://BioRender.com/n8vt282.

Microfluidic technologies are beginning to compete with traditional PDEV isolation for their ability to process samples quickly, with minimal sample volume, and to precisely control vesicle separation. The systems involve small channels and designed devices, such as 3D printed channels and microfluidic systems like Tesla, that separate vesicles based on parameters such as size, surface charge, and affinity interactions. The separation can be accomplished by controlled laminar flow, size-based filtration, and affinity capture in microchannels, depending on the device design. The level of control that can be achieved with microfluidic isolation of small sample volumes is one of the main attractions [70]. These relatively mild processing conditions retain the vesicle structure and biological activity, and the short processing time is an advantage over long centrifugation procedures. Some microfluidic systems can provide control over vesicle size distribution and cargo loading, which may be useful for applications requiring rapid and controlled processing. Importantly, cargo-loading or encapsulation efficiency should be distinguished from isolation recovery and purification performance, as these metrics describe cargo incorporation rather than vesicle isolation. However, there is still insufficient evidence that microfluidics has completely replaced traditional isolation techniques. Comparisons between platforms may be difficult due to deviations in device geometry, surface properties, flow conditions, and capture mechanisms. However, the smaller process capacity of many microfluidic systems remains a challenge for large-scale PDEV production. This standardization and scale-up remain significant impediments to their broader use in industry. However, the versatility of microfluidic platforms, in terms of their ability to integrate isolation and downstream processing, their compatibility with automation, and their suitability for large-scale screening, makes them an attractive target for future development. Their greatest potential could be in applications where large capacity methods like ultrafiltration are not required, but where large PDEV samples must be processed rapidly, yet with control and selectivity [71]. Figure 6 shows the main characteristics of advanced PDEV isolation technologies.

Because isolation studies differ substantially in plant source, starting material, processing conditions, and definitions of recovery and purity, direct numerical comparison across methods remains difficult. The practical performance of each approach therefore depends on the intended application and experimental conditions. Table 3 summarizes the principal trade-offs among recovery, purity, processing time, scalability, cost, and preservation of vesicle integrity to facilitate method selection.

### 4.3. Critical Challenges

PDEVs are being more widely used as natural nanocarriers; one of the primary sources of variation among studies is isolation. Variations in the extraction method, tissue type, centrifugation conditions, filtration, and purification processes may significantly affect vesicle recovery and the vesicle content in the final preparation [72]. Thus, the reported differences between PDEV studies may be due to variability in plant biological sources and methodological differences in the isolation process. Yield and purity are often conflicting. Repeated ultracentrifugation or enzyme pretreatment may be beneficial for recalcitrant plant materials such as roots or fibrous materials, but can lead to longer processing times and decrease scalability [73]. On the other hand, techniques aimed at enhancing vesicle purity, such as Density Gradient Centrifugation and SEC, can reduce contaminant levels but may require additional processing and lead to vesicle loss. Optimization of the strategy will thus be dependent on the desired goal: recover the maximum amount of product, achieve a high level of purification, maintain the integrity of sensitive products, or prepare a product on a larger scale. Another challenge is contamination, as protein, polysaccharides, pectin, starch, secondary metabolites, and other nanoscale particles are present in the plant matrices in high abundance. A few of these molecules may not be removed from PDEV preparations during purification [73]. Also, when tissue is disrupted, plant-derived nanoparticles may be produced that are similar in size and physical characteristics to the PDEVs, making them difficult to distinguish and characterize. Special attention must be paid to this issue when biological activity is attributed to vesicles, but without adequate proof that the effect is due to the vesicles rather than to other vesicles or fractions found within them. Notably, there is no universal agreement on isolation and quality control criteria. The particle concentration, size distribution, molecular cargo, and biological properties of the resulting preparations may vary with preparation conditions, such as extraction buffers, temperature, centrifugal force, membrane characteristics, gradient composition, and storage conditions [26]. Combined workflows can improve purification, but there are no standard procedures for quantitatively comparing purification between studies or for reproducibility. Standardized, measurable quality-control strategies will need to be implemented to resolve these bottlenecks. Comparative studies should be conducted in the future using the same plant source and starting material, and the results should include recovery yield, particle concentration, particle size distribution, and contamination indicators. There is a need to use multimodal characterization through particle analysis, microscopy, surface measurements, and molecular profiling to differentiate vesicle populations from co-isolated plant components. Reproducibility should also be evaluated for each batch under defined processing and storage conditions, and biological activity should be assessed in standardized cell or disease models. Systematic comparison of recovery, purity, processing time, and preservation of biological activity of tangential-flow filtration and centrifugation-based technologies is recommended for scale-up. Common reporting criteria would enable quantitative comparison of isolation methods and provide practical guidelines for selecting workflows for the intended PDEV applications. The challenges show that there is no perfect isolation technique for all the PDEV applications. For routine laboratory-scale recovery, DUC can provide a practical starting point; DGUC may be preferred when higher purity is required; SEC is useful when preservation of sensitive cargo and vesicle integrity is prioritized; UF/TFF is particularly suited to concentration and larger-volume processing; and microfluidic approaches may be advantageous for rapid, small-volume, or highly controlled workflows. The appropriate method ultimately depends on the required balance among recovery, purity, processing time, scalability, cost, and preservation of vesicle integrity. Future progress will rely on the development of standardized, application-specific workflows that enable meaningful comparison of isolation performance across different plant sources and processing conditions [74].

## 5. Physicochemical Characterization and Analytical Techniques

**Figure 7:**
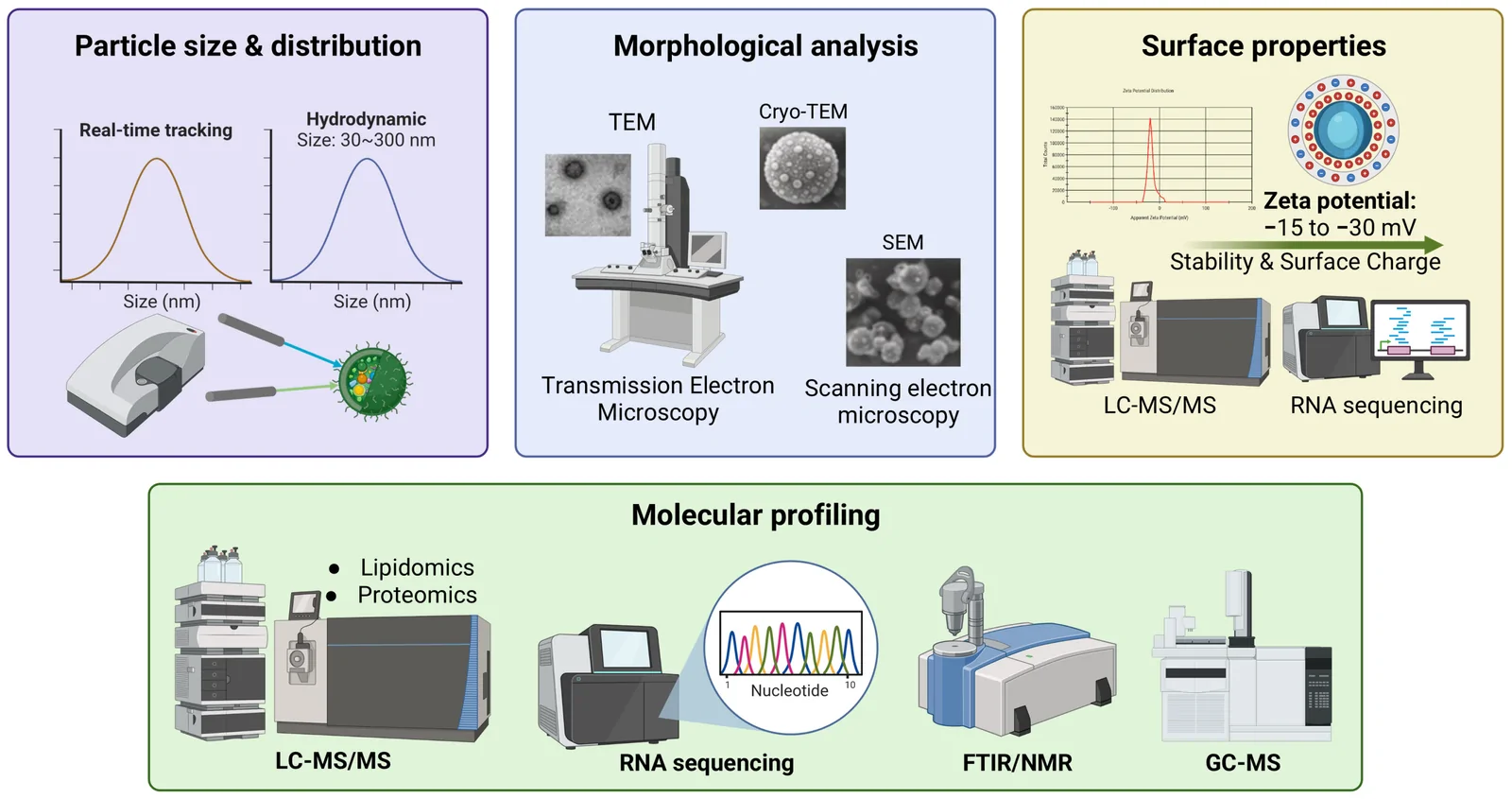
Advanced characterization of plant-derived extracellular vesicles (PDEVs). Purple, blue, yellow, and green panels show particle size, morphology, surface properties, and molecular profiling, respectively. Icons represent vesicles and analytical instruments; curves illustrate size, zeta-potential, and sequencing profiles. created in BioRender. Awlqadr, F. (2026) https://BioRender.com/r4d0lgu.

Plant-derived vesicles (PDEVs) can be found in preparations as vesicles of varying sizes, vegetative particles, and soluble contaminants, making reliable characterization more than just a particle-size measurement. Therefore, a combination of complementary analytical techniques is required to determine the particle size and size distribution, morphology, surface properties, and molecular composition of the particles. The selection of analytical methods should also depend on the objective of the study, because methods that provide rapid physicochemical measurements are not necessarily applicable for determining vesicular identity or biological composition. Dynamic light scattering (DLS) and nanoparticle tracking analysis (NTA) are two popular methods used for particle size analysis. DLS is a very quick method for measuring the hydrodynamic size of particles in suspension and is very convenient for routine assessment, but may be considerably affected by larger particles in heterogeneous samples. NTA, in contrast, uses particle-by-particle tracking and can provide information on particle concentration and size distribution, which is especially useful when there is population heterogeneity in PDEV [74]. Therefore, DLS and NTA are not techniques that can be used interchangeably; DLS is better suited for fast bulk measurements of particles, while NTA is better suited for obtaining higher-resolution measurements of more heterogeneous particle populations. Structural information unavailable with light-scattering techniques is obtained using microscopy. TEM can provide information on vesicle morphology and membrane structure, and cryo-TEM can provide near-native, hydrated TEM imaging with fewer structural artifacts from conventional sample preparation. SEM can be a helpful tool for understanding surface morphology and topology, but it is typically less informative for examining the internal structures of vesicles [64]. Morphological evidence alone is therefore most useful when used in conjunction with particle-size measurements. The surface charge (zeta potential) is another physicochemical parameter of importance to the interaction of vesicles with cells and to the stability of the colloid. PDEV preparations have a negative surface potential (although not all are equal, depending on plant source and isolation procedure). However, yam bean vesicles were reported to have a zeta potential of −26.5 mV, indicating relatively good dispersion stability [75]. Zeta potential, however, is insufficient to identify the vesicles or predict their biological performance, as the surface charge depends on the surrounding medium and experimental conditions. Molecular characterization provides data not available from particle-based and microscopic techniques. These technologies can be used to identify the identities and abundances of lipids and proteins in the sample, or small RNA populations and their potential regulatory functions, through LC–MS/MS and RNA sequencing, respectively [76]. FTIR can give information on the major functional groups associated with proteins, lipids, and nucleic acids, while NMR can yield information on lipid organization and metabolites. GC–MS is especially valuable for profiling readily volatilized and/or derivatizable secondary metabolites, including plant-derived classes. Such methods reflect varying levels of PDEV composition and should be chosen based on the molecular question at hand. The drawback is that none of these analytical techniques can fully describe PDEV preparation. Microscopy provides morphology but little molecular information; omics approaches offer molecular information, but not necessarily the enclosed molecules; and particle size measurements do not differentiate vesicles from similarly sized contaminants. A comprehensive characterization approach must therefore involve particle size, morphological imaging, surface analysis, and molecular profiling. The multimodal approach offers a more convincing identification of vesicles and enables a more confident correlation between physicochemical properties and cargo composition and biological activity [77]. Figure 7 is a summary of the complementary analytical approaches that were used for PDEV characterization.

For better reliability and comparability of PDEV studies, a minimum characterization framework should be taken into account when reporting plant-derived vesicular preparations. At least, characterization should encompass molecular cargo (if appropriate) as a surface or membrane associated component, particle concentration and size distribution, and morphological assessment; representative molecular cargo should be validated. Plant-derived preparations might also contain co-isolated protein aggregates, lipoprotein-like particles, polysaccharides, membrane fragments and other nanoparticles, so particle detection alone does not guarantee identification of a preparation as a vesicle. Therefore, the purity of the samples and the separation of the vesicular structures from non-vesicular contaminants should be assessed with appropriate negative controls and orthogonal analytical methods. Reporting of isolation conditions, particle concentration, size distribution, particle morphology, molecular composition and relevant purity indicators would also help to compare studies. While plant EVs are distinct from mammalian EVs in nature and composition, the use of MISEV principles may offer a framework for standardization of reporting and characterization. Such guidelines should focus on complementary measurements and not just one marker or analytical technique, and should also take into account the molecular and structural characteristics of the plant.

## 6. Stability, Bioaccessibility, and Gastrointestinal Fate

**Figure 8:**
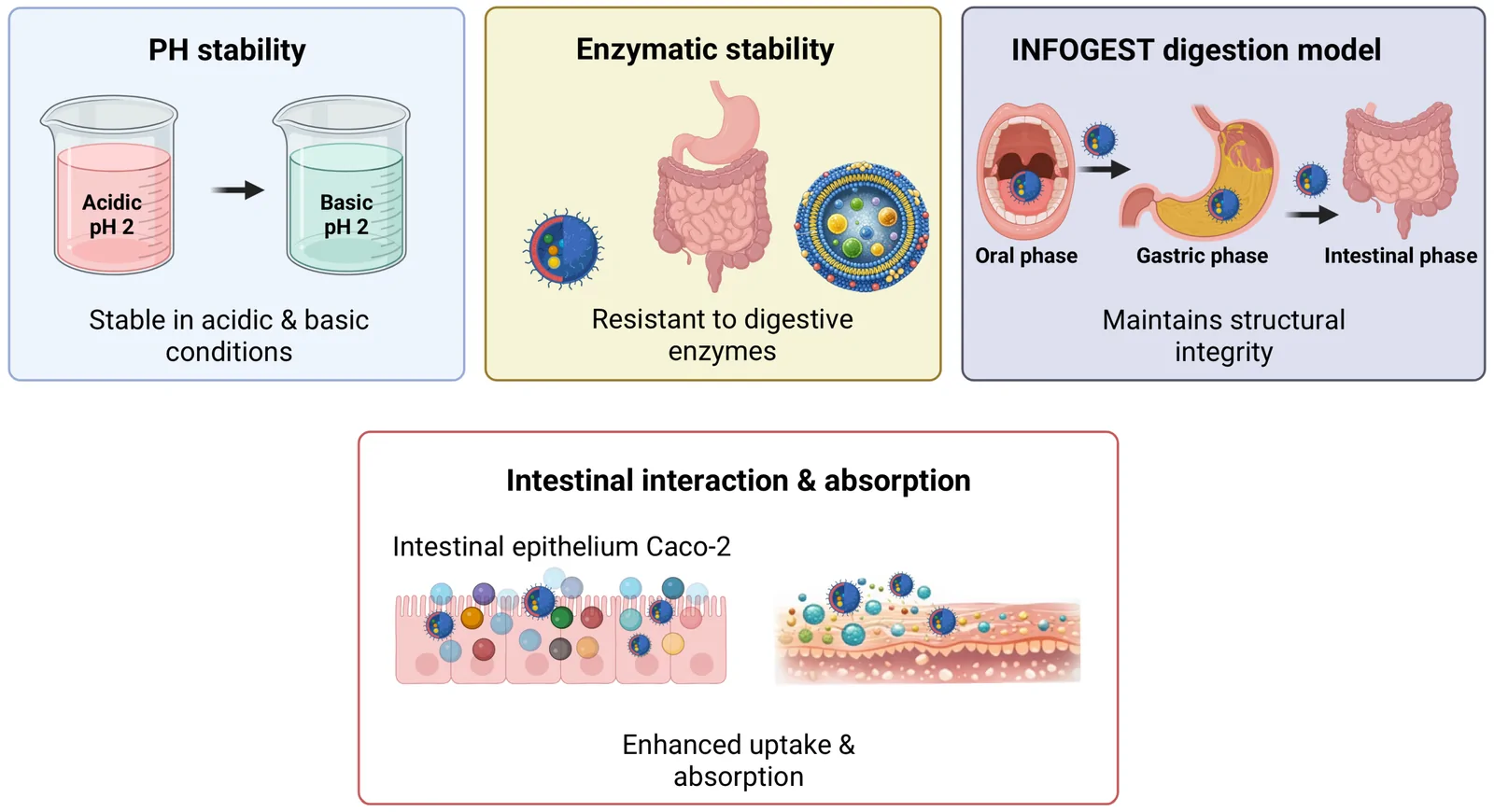
Stability, bioaccessibility, and gastrointestinal fate of plant-derived extracellular vesicles (PDEVs). Vesicle icons represent PDEVs, organ icons indicate digestive stages, and arrows show progression. Colored circles schematically depict vesicles and other components at the intestinal barrier and in circulation. Created in BioRender. Awlqadr, F. (2026) https://BioRender.com/2c7tegw.

Gastrointestinal stability is crucial to the potential of oral delivery of PDEVs. The lipid bilayer can protect vesicles and their cargo during digestion, but stability is preparation- and source-dependent. Catharanthus roseus-derived nanovesicles retained structural and biological properties under acidic and enzyme-containing conditions [78], while blueberry extracellular vesicles remained stable during simulated gastrointestinal digestion and were taken up by intestinal Caco-2 cells [32]. These findings support the oral-delivery potential of selected PDEV preparations, but they should not be generalized to all plant sources or isolation methods. Vesicle composition, processing, storage, and the digestion model can each affect aggregation, cargo retention, epithelial uptake, and biological activity. Therefore, gastrointestinal fate should be evaluated for each preparation using validated digestion and intestinal-cell models. The principal processes relevant to PDEV stability, bioaccessibility, and gastrointestinal fate are summarized in Figure 8.

More importantly, much of the evidence supporting the gastrointestinal stability is based on in vitro digestion models such as simulated gastric and intestinal digestion, which do not measure it in humans or live animals. These models give important information about the physicochemical stability but cannot completely mimic gastrointestinal mucus, peristalsis, immune interactions, enzymatic variability, or even the subsequent systemic absorption. Thus, these data should not be concluded as proof of complete in vivo stability or improved oral bioavailability when digesting vesicles intact and without breaking the vesicle structure or its contents. Additional in vivo research is needed to address what happens to these vesicles, how they release their contents, how they are absorbed in the intestine, how they are metabolized, and where they go in the body.

## 7. Engineering and Functionalization of PDEVs

### 7.1. Cargo Loading Strategies

Efficient cargo loading is an important step in the engineering of PDEVs to improve their effectiveness as targeted delivery systems for bioactive agents. Several methods have been developed for loading vesicles with cargo, and many have been used to load cargo into PDEVs. The three most well-known of these methods are passive loading, electroporation (EP), and sonication, and each offers unique advantages based on the cargo’s prevailing physicochemical properties and the intended function when enclosed within the PDEVs.

These methods vary greatly in their underlying loading mechanisms, meaning each approach will enable efficient loading of a different type of cargo. Passive loading is primarily based on diffusion, hydrophobic interactions, and partitioning into and/or across the vesicular lipid bilayer. Therefore, relatively small, membrane-permeable molecules, especially hydrophobic ones, are best suited for this, whereas larger, highly polar molecules and nucleic acids are less effective. Electroporation is a process that temporarily weakens the membrane, making it more permeable to charged, hydrophilic macromolecules (e.g., siRNA, miRNA, and other nucleic acids) to enter the vesicle lumen. In contrast to sonication, which produces mechanical shear and acoustic cavitation to temporarily break open the vesicular membrane, allowing small molecules and hydrophobic compounds to enter, this process does not produce mechanical shear. Depending on the energy used, treatment time, and vesicle composition, the extent of damage to the membrane can vary, making optimization of the process very important to ensure vesicle integrity [79].

**Figure 9:**
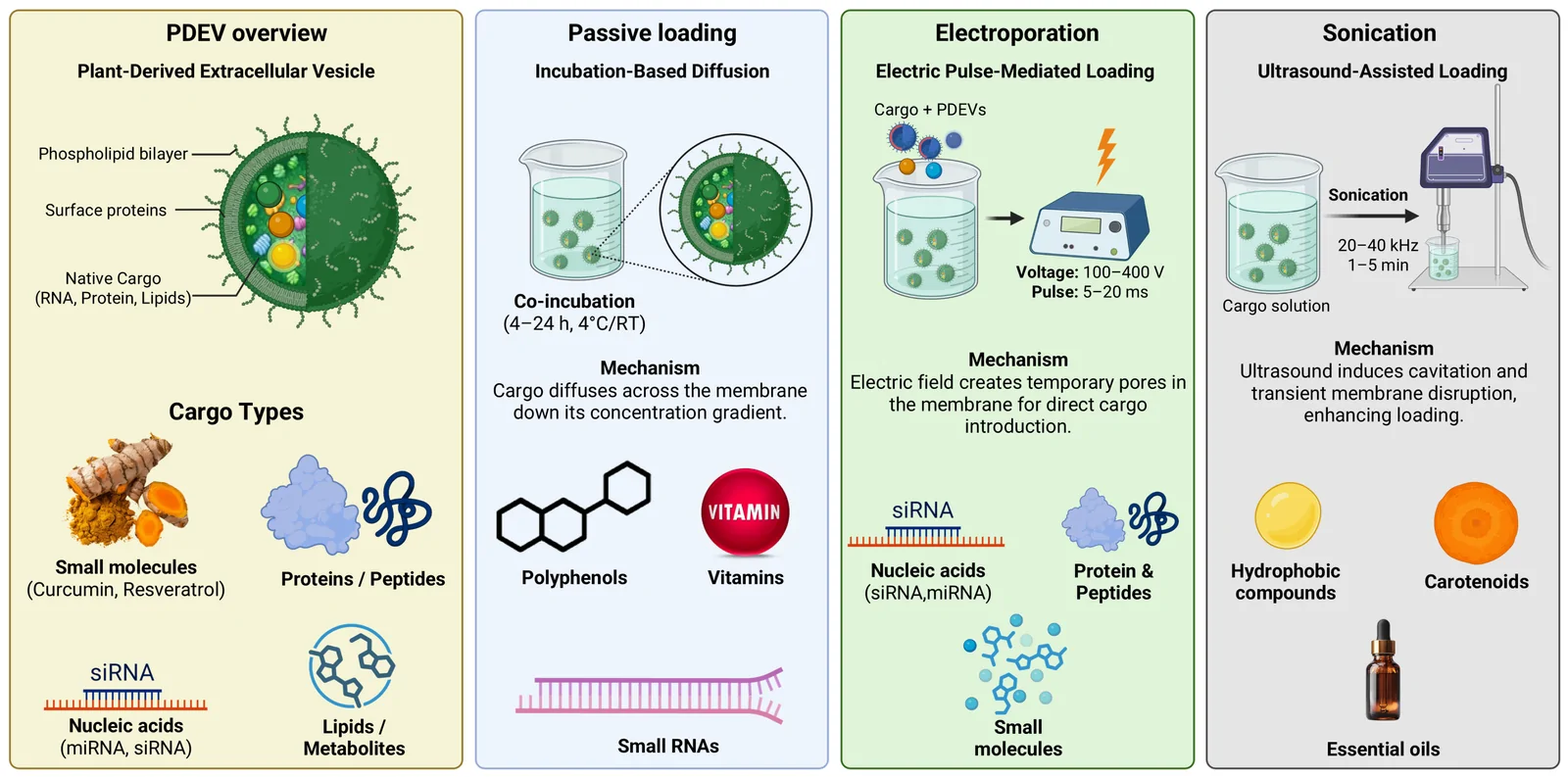
Cargo loading strategies for plant-derived extracellular vesicles (PDEVs). Passive loading involves cargo diffusion during co-incubation; electroporation uses electrical pulses to create transient membrane pores; and sonication uses ultrasound to facilitate cargo entry. Icons illustrate PDEV structure and representative cargo, including small molecules, proteins, nucleic acids, and lipids. Arrows indicate processing steps, and the lightning bolt denotes electrical pulses. Created in BioRender. Awlqadr, F. (2026) https://BioRender.com/05hkgpb.

Passive loading is a relatively simple and biocompatible method of loading cargo into PDEVs that utilizes diffusion or incubation of bioactive molecules with PDEVs under controlled conditions. For example, when producing vesicles from plant sources such as *Nauclea officinalis* or tomato, the isolated vesicles can be incubated with a variety of compounds, including hydrophobic compounds derived from Andrographis paniculata and small-molecular-weight compounds such as tetrahydrocannabinol, at temperatures ranging from 4 to 25 °C for several hours to overnight. This process can result in spontaneous incorporation of cargo into PDEVs through concentration gradients and lipid affinity. In a study by Peng et al., PLC-2P2 loaded onto PDEVs during passive incubation exhibited greater cellular targeting and bioavailability than its free counterpart [80]. The disadvantage of passive loading is that encapsulation efficiency is typically low (<20%), thereby limiting the ability to encapsulate nucleic acids or large biomolecules [81]. EP is widely utilized to load nucleic acids (e.g., miRNA, siRNA, or CRISPR components) into vesicles. In this method, very short electrical pulses (approximately 100–400 V, typically in the millisecond range) are used to generate temporary disruptions in the lipid bilayer of PDEVs, thereby promoting cargo entry or uptake into their interiors. Electroporation has been investigated as a cargo-loading strategy for extracellular vesicles, particularly for the incorporation of nucleic acids into mammalian exosome systems [82]. These studies demonstrate the general feasibility of electroporation for vesicular cargo loading; however, their findings should not be considered direct evidence of equivalent loading efficiency, membrane integrity, or biological performance in PDEVs. The application of electroporation to PDEVs therefore requires plant-specific optimization and experimental validation [83]. Although electroporation can yield high-efficiency delivery systems, if not optimized, it may cause vesicle aggregation and/or RNA degradation. Sonication (also known as ultrasonication) is a mechanical loading technique that uses shear forces generated by ultrasonic waves (most commonly at 20–40 kHz) to transiently disrupt vesicle membranes, thereby increasing loading efficiency for both hydrophobic drugs and small-molecule formulations. For example, the encapsulation efficiency of tomato-derived extracellular vesicles using sonication was 47% compared to 15% for passive loading methods, resulting in a significant improvement in anti-cancer activity and intracellular delivery [81]. Furthermore, when sonication is used, drug encapsulation can be enhanced while preserving the vesicles’ structural integrity; however, excessive energy input during sonication may alter the vesicles’ integrity and biological function [84]. Therefore, the appropriate choice of cargo loading should depend on the cargo’s physicochemical properties and its intended use. Passive loading is useful for low payloads (with relatively low loading efficiency) and when structural maintenance and ease of processing are concerns for the cargo. Electroporation can facilitate the loading of nucleic acids and other charged macromolecules by temporarily increasing membrane permeability; however, its loading efficiency and effects on vesicle integrity depend on the electrical conditions, cargo, and vesicle composition. Excessive electrical conditions can cause vesicle aggregation or cargo degradation. Sonication can yield higher loading efficiency for small molecules and hydrophobic compounds, so long as the acoustic energy is not excessive enough to destroy membrane integrity and biological activity. Therefore, the loading efficiency cannot be viewed in isolation from cargo stability, vesicle integrity, and downstream biological activity. Hence, the selection of the loading strategy should be based on the stability of the loading, the integrity of the vesicles, and the biological activity downstream [85]. PDEVs have been investigated for cargo loading through passive loading and sonication, while electroporation represents a potential loading strategy that requires further plant-specific optimization and validation. The suitability of each approach depends on cargo type, loading efficiency, vesicle integrity, and intended delivery function. Figure 9 summarizes the principal cargo-loading strategies used for PDEVs.

### 7.2. Surface Engineering

**Figure 10:**
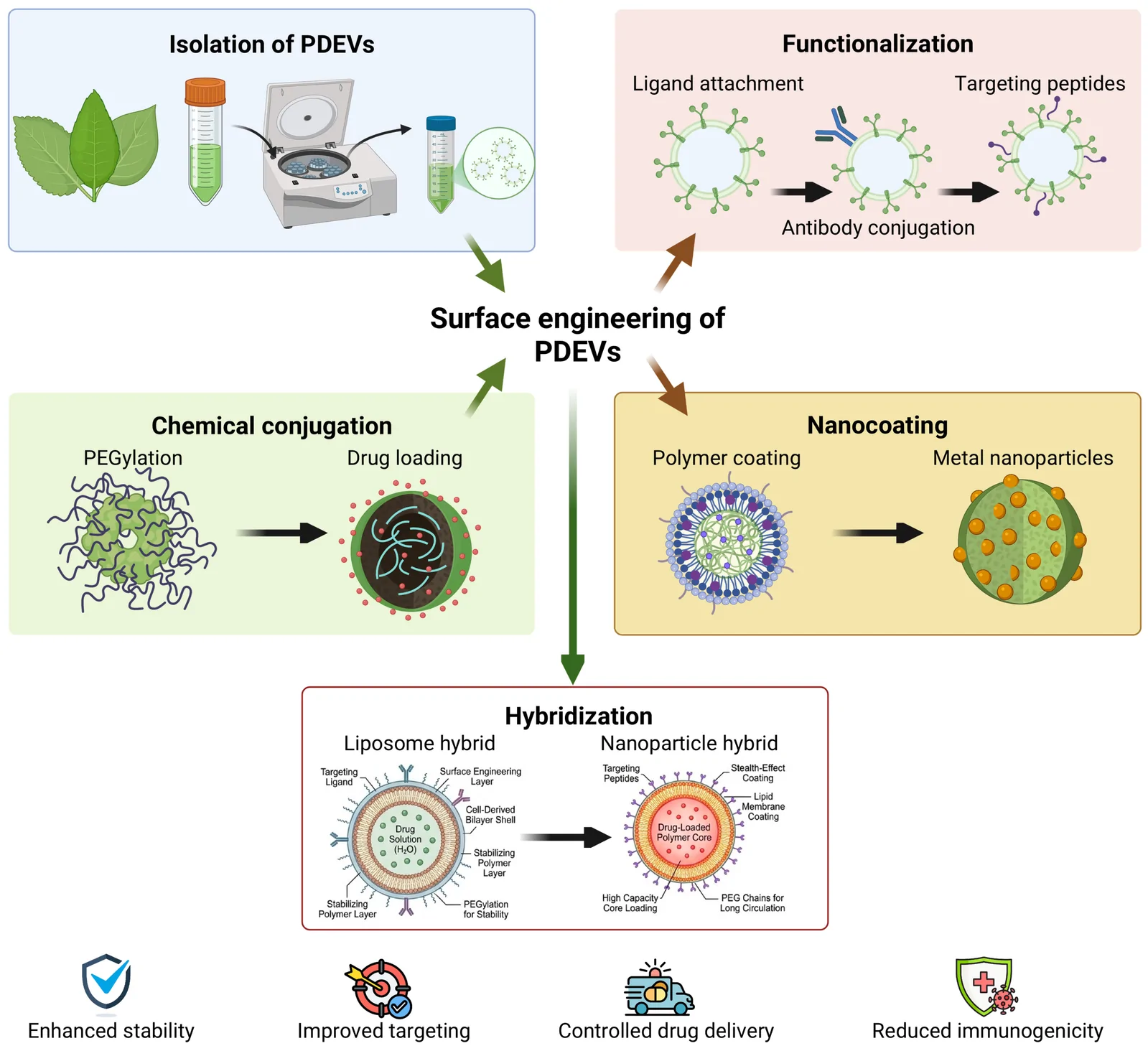
Surface engineering strategies for plant-derived extracellular vesicles (PDEVs), including ligand, antibody, and peptide functionalization; chemical conjugation; nanocoating; and hybridization with liposomes or nanoparticles. Arrows indicate isolation steps and connections between engineering approaches; arrows within panels link representative modifications. Bottom icons depict intended benefits: enhanced stability, improved targeting, controlled drug delivery, and reduced immunogenicity. Created in BioRender. Awlqadr, F. (2026) https://BioRender.com/vkwwppr.

Surface engineering is an emerging approach for modifying the targeting, stability, and delivery behavior of PDEVs. This is accomplished through the following key methods: ligand conjugation, PEGylation, and target-specific functionalization, which together modify the native vesicle into a precision nanocarrier. Ligand conjugation is one of the most common methods for achieving selective cellular targeting by attaching bio-recognition molecules (such as peptides, antibodies, or aptamers) to the vesicle surface. For example, exosome-like vesicles derived from Cucurbita moschata that have been functionalized with aptamers exhibited greatly enhanced uptake in EpCAM-positive prostate cancer cells, leading to better tumor suppression and less off-target toxicity as compared to non-functionalized vesicles. Peptide-modified mammalian exosomes have demonstrated enhanced affinity toward specific cell types and facilitated the delivery of therapeutic agents such as siRNA and gene-editing platforms [86,87]. These findings provide comparative evidence for receptor-mediated targeting but do not establish equivalent targeting efficiency or intracellular delivery performance in PDEVs. PEGylation (polymer modification with polyethylene glycol) is a vital technique for enhancing the pharmacokinetic properties of PDEVs. In general, the entrapment of PEG molecules on the vesicle membrane helps prevent their aggregation, reduces recognition by the mononuclear phagocyte system, and increases their systemic circulation time. Recent research demonstrates that PEGylated or biomimetic exosomal systems exhibit greater overall stability, lower immunogenicity, and greater biodistribution potential than unmodified systems [88]. The ability to couple PEGylation with ligand conjugation means a delivery platform/system can now effectively possess both stealth and target-specific features, thereby improving delivery efficiency in complex biological environments. The selection of surface-engineering techniques will depend on the planned delivery system, target cell, and the desired pharmacokinetic properties. Ligand conjugation is especially useful when selective recognition of a specific receptor or surface marker is required, and PEGylation is primarily used to increase colloidal stability and extend circulation time by minimizing nonspecific interactions. Using PEGylation in conjunction with targeting ligand(s) may provide complementary stealth and targeting roles, but might result in excessive surface modification, which could affect membrane properties or the interaction between ligand and receptor. To optimize surface engineering, a balance should be struck between targeting specificity, vesicle stability, cellular uptake, and preservation of the biological properties of PDEVs [89]. Target-specific delivery of engineered systems results from the combination of ligand functionalization and advanced nanotechnology, enabling delivery to specific tissues or cells. For example, engineered extracellular vesicles displaying targeting peptides or antibodies can selectively accumulate in diseased tissues, such as cancerous tissues, cartilage, or inflamed vascular structures. For instance, the use of engineered chondrocyte-affinity peptide extracellular vesicles has resulted in efficient and localized delivery of the CRISPR/Cas9 gene-editing system; both gene editing and therapeutic efficacy were substantially improved in animal models of osteoarthritis. Passively targeted plant-derived vesicles have also demonstrated improved delivery of therapeutic agents and RNA molecules, as well as functional modulation of recipient cells. This could lead to potential treatment applications for different disease conditions, including anti-inflammatory and metabolic disorders [80]. Overall, Surface-Engineered PDEVs offer synergistic advantages, including enhanced cellular uptake, increased protective/defensive properties of the delivered therapeutic agents, and controlled biodistribution. These properties are especially useful when considering how we can use food products to provide health benefits through biotechnology. The ability to deliver bioactive compounds accurately to the intestines or other parts of the body is an important aspect of functional foods. Another reason delivering bioactive compounds in functional foods using plant vesicles makes a lot of sense is that these vesicles have low immunogenicity, meaning they are safe for people to eat. Surface engineering of PDEVs through ligand conjugation, PEGylation, targeting peptides, nanocoating, or hybrid nanostructures can enhance cellular uptake, circulation stability, targeted delivery, and therapeutic performance. Figure 10 summarizes the principal surface-engineering strategies used for PDEVs.

### 7.3. Hybrid Systems

The combination of PDEVs with advanced nanomaterials is a promising strategy for improving stability, targeting, and controlled release of functional foods and biomedical products. The most promising methods involve using PDEVs in combination with either biopolymer matrices or nanoemulsions. The use of PDEVs in combination with nanoemulsions enables efficient delivery of hydrophobic bioactive compounds by increasing their loading capacity and enhancing delivery efficiency. Surfactants typically stabilize the oil–water interface in an oil–water-based nanoemulsion (i.e., surfactant-stabilized oil–water nanoemulsion), which allows for the incorporation of lipophilic compounds into the PDEV membrane. There is growing evidence that fusing or co-embedding plant exosomes with synthetic liposomes or lipid-based nanoemulsions significantly increases exosomal membrane fluidity, cargo encapsulation efficiency, and cellular uptake. Hybridization of extracellular vesicles with liposomal systems has been investigated to improve cargo delivery and tissue interactions; however, evidence from non-plant extracellular vesicle systems should be considered comparative rather than direct validation of equivalent performance in PDEVs [90]. Furthermore, ginger-derived extracellular vesicle-like nanoparticles loaded with indocyanine green exhibited improved photothermal stability, enhanced reactive oxygen species generation, and equivalent tumor inhibition, thereby illustrating the synergistic interaction between plant vesicles and nanosized lipids [91]. The results of this research suggest that using nanoemulsion-hybridized systems improves both the physical and chemical stability and the functional bioactivity of PDEVs. The principles behind the mechanisms of these hybrid systems are related to the characteristics of the incorporated material. The oil-rich environment of nanoemulsions can enhance the dispersion and incorporation of hydrophobic compounds, and interactions among lipid components can create associations between the cargo and the vesicular membrane. However, polymeric matrices can provide a physical barrier around PDEVs, which may help limit their exposure to environmental stresses and control the diffusion of cargo associated with the vesicles. These differences are particularly relevant when delivering poorly water-soluble compounds, for which nanoemulsion-based systems may facilitate solubilization and delivery, whereas hydrogels and other polymer-based systems may be more suitable when prolonged retention and sustained release are desired [92]. Hybridized PDEVs with a biopolymer matrix (i.e., hydrogels, fibrin, gelatin, or polysaccharides) have shown improved structural stability, sustained release, and targeted delivery. Biopolymers act as 3-dimensional scaffolds around the vesicles, protecting them from degradation while providing controlled kinetics for their eventual release. Fibrin-based hydrogel systems incorporating extracellular vesicles have also been investigated for sustained release and tissue-repair applications; however, studies using non-plant extracellular vesicles should be distinguished from PDEV-based systems [93,94]. Plant-derived vesicles have also been incorporated into hydrogel-based systems for experimental delivery applications; however, the effects on retention, release behavior, and biological activity depend on the specific vesicle preparation and hydrogel formulation [95]. Another advanced example is thermo-responsive hydrogel–PDEV composites, which have demonstrated that drug-loaded vesicles released 94% of their contents over a 5-day period, allowing for extended therapeutic activity while also impeding tumor progression [96]. Therefore, the selection of a hybrid system should consider the intended delivery system and release profile. Nanoemulsion-based systems are better suited for enhancing the solubilization and delivery of hydrophobic compounds, whereas in situ-based systems offer extended retention and controlled release in a localized area. Various properties, such as enhanced surface functionalization or shielding of the vesicular part, can also be introduced through hybridization with inorganic nanostructures. Adding complexity to the structure, though, can also raise other concerns about material compatibility, process reproducibility, characterization, and safety. Therefore, hybridization should be planned to achieve a specific functional benefit, rather than merely adding more structures to the delivery vehicle [97,98]. Additionally, hybridizing polymeric and inorganic nanostructures yields more functional materials than non-hybridized forms. For instance, Cucurbita-derived exosomal coatings on metal–organic framework (MOF) nanoparticles have been reported to confer immune evasion, confer hemocompatibility on the coated particles, and enhance targeted drug delivery for treating cancer [99]. Therefore, PDEVs have the potential to serve as both biomimetic coatings and active delivery platforms for therapeutic agents.

## 8. Applications in Next-Generation Promotion of Health

### 8.1. Delivery of Bioactive Compounds

PDEVs offer flexible platforms for the delivery of bioactive compounds such as polyphenols, flavonoids, vitamins, carotenoids, and other phytochemicals, which are potentially useful for functional food, nutraceutics, and therapeutics applications. They have a vesicular structure that allows them to carry various cargo molecules and can also be enriched with bioactive molecules that are not easily soluble and deliverable in their individual form. Polyphenols have been among the most widely studied compounds for delivery via PDEV. For instance, curcumin has been encapsulated in a plant-based nanovesicle to enhance its delivery and biological activity. The efficiency of curcumin entrapment in extracellular vesicles isolated from Lentinula edodes has been reported to be approximately 69%, resulting in greater intestinal retention and microbiota-associated biotransformation [96]. Curcumin has also been loaded into tomato, ginger, and banana vesicles, showcasing the versatility of PDEV-based systems for various applications, including nutraceutical and colon-targeting. These studies demonstrate the potential of PDEV-based formulations to facilitate the delivery of phytochemicals to specific biological sites; however, the extent of targeting and delivery efficiency depends on the plant source, isolation procedure, cargo, formulation, and experimental model. Bioactive molecules of different classes can also be packed into the same PDEVs. The co-delivery of exogenous components, such as lipids, miRNAs, and phytochemicals, along with endogenous components, such as vesicles, may create multifunctional delivery systems in which all components contribute to the overall biological response. For example, dark tea-derived nanovesicles, which contain flavonoids and phenolic acids, have been shown to alter gut microbiota composition and immune responses, and to release antioxidants [99]. Such results demonstrate the potential of plant-derived natural vesicles as a means of transport and as an integrated system comprising several complementary components. In addition to small-molecule phytochemicals, PDEVs have been studied for the delivery of macromolecules, such as nucleic acids. Ginger vesicles loaded with siRNA and 6-shogaol have been developed to deliver siRNA to the colon, and grapefruit vesicles loaded with siRNA have been delivered to the cytoplasm and suppressed genes [100]. These findings provide experimental evidence for nucleic-acid delivery, but the reported outcomes are model-dependent and should not be interpreted as evidence of established systemic delivery or therapeutic efficacy in humans. However, vesicles derived from citrus fruits containing SARS-CoV-2 S1 mRNA triggered systemic and mucosal immune responses, and grapefruit- and mandarin-derived vesicles containing vaccine-associated antigens resulted in higher serum IgG responses when compared with antigen alone. These studies demonstrate the versatility of PDEV cargo capacity and its utility beyond standard nutraceutical delivery. PDEV-based delivery can also be used alongside targeting and external loading strategies. In fact, ginger-derived vesicles have been studied for receptor-mediated targeting to macrophages, and curcumin-loaded vesicles have been explored for inflammatory and colonic disorders [101]. Based on the current evidence, PDEV-based systems can accommodate a wide variety of bioactive compounds and biomolecules with potential applications in functional foods, nutraceutics, and therapeutic delivery systems. A summary of the representative delivery strategies and reported outcomes is presented in Table 4.

### 8.2. Nutrients in Diet

The presence of PDEVs in edible plants could link nutrition to nanoscale biological delivery. These vesicles are naturally found to contain various combinations of lipids, proteins, nucleic acids, metabolites, and phytochemicals, which may be responsible for the biological activities observed in plant-based foods. Their molecular composition depends on their host plant and processing methods, as evidenced by the differences in metabolite, lipid, and protein composition between extracellular vesicles from Portulaca oleracea fresh and dried plants [108]. When considering PDEVs as components of functional foods, it is important to account for their compositional differences, as food processing can affect the vesicles’ characteristics and their associated bioactivity. Naturally occurring phytochemicals and regulatory molecules are especially discussed about the nutritional relevance of PDEVs. The plant-derived vesicles from edible plants and beverages may contain polyphenols, flavonoids, terpenoids, ceramides, and other metabolites that could be nutritionally significant. The vesicles of *Eutrema japonicum* elicited immunostimulatory activity and were used to investigate their use as oral nutraceutical systems with ceramides, naringenin, and genistein [109]. Likewise, extracellular vesicle-like nanoparticles containing mature miRNAs have been reported in coffee beverages, suggesting that beverages, beyond their nutritive components, can also supply vesicle-associated nucleic acids. Plant-derived miRNAs have also attracted interest because of their proposed role in dietary cross-kingdom communication. Some experimental studies have reported biological effects associated with soybean-derived miRNAs and plant miRNA159 in mammalian cellular models, including effects on cell proliferation, apoptosis, and oncogenic signaling pathways [110]. However, these findings should not be interpreted as establishing dietary miRNAs as confirmed regulators of mammalian gene expression in vivo. Detection or cellular uptake of plant-derived miRNAs does not necessarily demonstrate functional intracellular delivery or sequence-specific gene regulation, and their physiological relevance may depend on dose, gastrointestinal stability, absorption, tissue exposure, and intracellular concentration. Based on these observations, it can be concluded that nucleic acids that are part of the dietary vesicles could constitute a further molecular component by which plant foods can interact with the mammalian biological system. Nevertheless, the proportion and physiological relevance of dietary miRNA transfer are important considerations when studying this suggested mode of action. By associating naturally occurring nutrients with vesicle-associated bioactive molecules, PDEVs can contribute to the development of functional foods and precision nutrition strategies. For instance, vesicles extracted from grapefruit and tomato produce have been explored as functional food ingredients and delivery systems, and differences in vesicle yields and loading efficiencies have been reported across plant sources. Other food-origin vesicles that have been evaluated are for intestinal protection, antioxidant activity, and metabolic support, such as vesicles generated from *Ulva lactuca*, blueberry, *Opuntia ficusindica*, and *Eruca sativa* [11]. In summary, the nutritional potential of PDEVs derives from their intrinsic molecular content, food-source-dependent composition, and compatibility with food applications. Although they can be used in functional food systems, there are still differences between the plants from which they are derived, processing, composition, and biological response that should be taken into consideration when developing these systems. The representative applications of PDEVs in dietary nutrition and functional foods are summarized in Table 5.

### 8.3. Probiotics and Synbiotics Delivery

Functional foods incorporating PDEVs have been investigated as an emerging approach for microbiome-targeted nutrition. However, current evidence primarily concerns the ability of PDEVs or their associated cargo to interact with and modulate the gut microbial environment rather than their established ability to encapsulate, protect, and deliver viable probiotic organisms. Therefore, gastrointestinal stability and microbiota modulation should be distinguished from direct probiotic delivery. PDEVs may interact with intestinal microorganisms and influence host–microbial interactions and gut homeostasis; however, these effects should be distinguished from those mediated by bacteria-derived extracellular vesicles or other microbial vesicular systems [117]. PDEVs may instead act as potential modulators of microbial activity through their associated lipids, miRNAs, metabolites, and other molecular components. Such effects represent microbiota modulation or potential prebiotic-like activity rather than direct delivery of viable probiotic organisms. In particular, plant-derived miRNAs have been investigated for their potential effects on gut microorganisms. For example, miR2911 has been reported to remain relatively stable during digestion and to promote Bifidobacterium growth in an experimental model, suggesting a potential prebiotic-like effect [118]. Likewise, garlic-derived vesicles have been associated with increased abundance of B. thetaiotaomicron and reduced inflammation in colitis models. These findings support the potential of plant-derived vesicles to modulate the gut microbial environment, although the relative contributions of intact vesicles, vesicle-associated cargo, and direct microbial interactions remain to be established. Accordingly, these studies should not be interpreted as evidence that PDEVs function as established carriers for viable probiotic organisms. PDEV-based systems can impact the composition of microflora and production of beneficial metabolites, including short-chain fatty acids (SCFAs), which can contribute to intestinal, metabolic, and immune health [119]. The question, though, of whether the effects are caused by the vesicles themselves, their molecular contents, or their interactions with microorganisms, is of great interest for future research. In conclusion, PDEVs may have potential for incorporation into probiotic and synbiotic formulations through their bioactive cargo and microbiome-modulating properties. However, prebiotic-like microbiota modulation, delivery of microbial metabolites, co-formulation with viable probiotics, and bacteria-derived extracellular vesicles represent distinct concepts and should not be considered interchangeable. Direct evidence that PDEVs preserve probiotic viability or enhance probiotic colonization remains limited. Future studies should therefore distinguish these applications and evaluate vesicle composition, microbial interactions, gastrointestinal stability, probiotic viability where applicable, and biological efficacy. Table 6 presents representative applications of PDEVs relevant to probiotic and synbiotic strategies.

### 8.4. Anti-Cancer Properties

The use of PDEVs has gained traction as a new opportunity for the prevention and treatment of cancer due to their natural bioactivity, ability to deliver specifically, and potential to alter numerous oncogenic pathways. PDEVs are nanosized vesicles that contain a variety of active biological molecules, such as microRNAs (miRNAs), proteins, lipids, and phytochemicals, that contribute to their anti-cancer effects. PDEVs are not only more biocompatible, less toxic, and more stable than traditional chemotherapeutic agents, but they also represent strong candidates for the development of functional foods as cancer therapies. One of the major mechanisms through which PDEVs exert anti-cancer effects is by inhibiting tumor growth and inducing apoptotic cell death. Dark tea-derived exosome-like nanoparticles have been shown to cause a significant reduction in tumor growth by suppressing the Akt signaling pathway, decreasing proliferation markers (e.g., NF-κB and cyclin D1), and activating tumor suppressor pathways (e.g., p53 and caspase-3) in the context of hepatocellular carcinoma [126]. In addition, *Platycodon grandiflorum*-derived vesicles have been found to increase reactive oxygen species (ROS) production in tumor cells, thereby causing apoptotic cell death and inhibiting tumor growth in models of breast cancer. It is essential to activate the immune system to combat tumor immune evasion, with consequent improvements in long-term therapeutic outcomes. PDEVs can modulate the composition of the cytokines released from macrophages by inducing them to differentiate into the M1 subtype and enhancing their production of specifically pro-inflammatory cytokines [127]. A second way that PDEVs exert their anti-cancer effects is by modifying the gut microbiota and overall metabolism. The use of plant-derived nanoparticles, such as those derived from ginger, can positively influence the composition of the gut microbiota and lead to increased production of metabolites that promote tumor suppression, as well as improve cancer treatment through immunotherapy [128]. Similarly, vesicles derived from garlic have been shown to alter the gut microbiota and decrease inflammation, providing additional support for the role of these vesicles in cancer prevention [127]. In addition to these effects, PDEVs can serve as advanced drug delivery systems for cancer treatment. These engineered plant-derived vesicles can be loaded with chemotherapeutic agents, like doxorubicin, and modified with targeting ligands to optimize their uptake at the tumor site and decrease systemic toxicity [129]. PDEVs’ ability to penetrate biological barriers and deliver bioactive agents to target sites enables them to achieve significantly greater therapeutic efficacy than traditional delivery systems. As shown in Table 7, PDEVs exhibit anti-cancer potential through multiple mechanisms, including inhibition of tumor growth, induction of apoptosis, modulation of oncogenic signaling pathways, immune regulation, and engineered chemotherapeutic delivery.

### 8.5. Anti-Inflammation and Antioxidant Properties

PDEVs have been investigated for antioxidant, anti-inflammatory, and tissue-repair effects, but the evidence is preparation- and model-specific. Walnut exosome-like nanoparticles reduced oxidative and inflammatory markers [136], whereas cardoon-derived extracellular vesicles reduced ROS and nitric oxide and activated SIRT1/AMPK-associated metabolic signaling [137]. Prunus mume vesicle-like particles inhibited NEK7-NLRP3 interaction and inflammasome activation [138]. Artemisia argyi-derived nanovesicles inhibited the TLR4/NF-kappaB/NLRP3 axis and activated Nrf2-associated antioxidant signaling [139]. Coptis chinensis nanovesicles promoted M2-like macrophage polarization and wound repair [140], and coix seed-derived exosomes reduced pro-inflammatory mediators in an ulcerative-colitis model [141]. Fermented Hordeum vulgare nanovesicles also reduced inflammatory mediators in cell models [142]. In addition, polyphenol-loaded plant extracellular vesicles have been evaluated for neuroprotective delivery through the microbiota–gut–brain axis [143]. Together, these studies support the therapeutic potential of selected PDEVs while underscoring the need to avoid extrapolating findings across plant sources, cargos, doses, and disease models. The principal anti-inflammatory applications are summarized in Table 8.

### 8.6. Critical Comparative Framework for PDEV Engineering and Translation

Across the applications discussed above, the reported performance of PDEVs should be interpreted in relation to the biological source, preparation and purification workflow, vesicle identity and quality, cargo composition, engineering strategy, administered dose, route of administration, and experimental model. Differences in reported delivery efficiency, biological activity, stability, and targeting may therefore reflect methodological differences as well as intrinsic properties of the vesicles. Direct comparison of individual outcomes is consequently difficult when these variables are not standardized. A meaningful assessment of engineered PDEV systems should consider the relationship between plant source, preparation method, physicochemical and molecular characterization, cargo composition and loading, surface modification or hybridization, dose and administration route, biological model, and quantitative outcome. In particular, engineering studies should report parameters such as vesicle recovery, loading efficiency, cargo retention, membrane integrity, cellular uptake, release behavior, biodistribution, and biological efficacy where applicable. These measurements provide a more informative basis for comparison than reporting cargo incorporation or enhanced cellular uptake alone. The biological model and exposure conditions are equally important when interpreting PDEV performance. Results obtained from in vitro systems cannot necessarily be compared directly with animal studies, while increased cellular uptake does not by itself demonstrate improved biodistribution or therapeutic efficacy. Similarly, biological effects following oral administration do not establish that intact PDEVs reach a specific target tissue. Dose, administration route, exposure duration, disease model, and analytical endpoint should therefore be considered when interpreting reported biological effects. Importantly, engineering feasibility should be distinguished from translational validation. Successful cargo incorporation, surface modification, or enhanced cellular uptake does not necessarily establish targeting specificity, pharmacokinetic behavior, safety, or therapeutic benefit. Conversely, biological activity observed from a PDEV preparation does not always demonstrate that the vesicular structure itself is responsible, because associated or co-isolated molecular components may contribute to the observed effect. Appropriate unmodified-vesicle controls, free-cargo controls, defined doses, quantitative characterization, and reproducible biological endpoints are therefore important for establishing causal relationships. Accordingly, the critical contribution of this review is to organize the available PDEV evidence according to the interconnected factors of source, preparation, vesicle quality, cargo, engineering strategy, exposure conditions, biological model, quantitative outcome, and evidence level. This framework distinguishes experimentally demonstrated PDEV performance from preliminary findings and extrapolations from other extracellular-vesicle systems, while identifying the methodological variables that require greater standardization for reliable cross-study comparison and future translational development.

## 9. Future Perspectives and Emerging Trends

**Figure 11:**
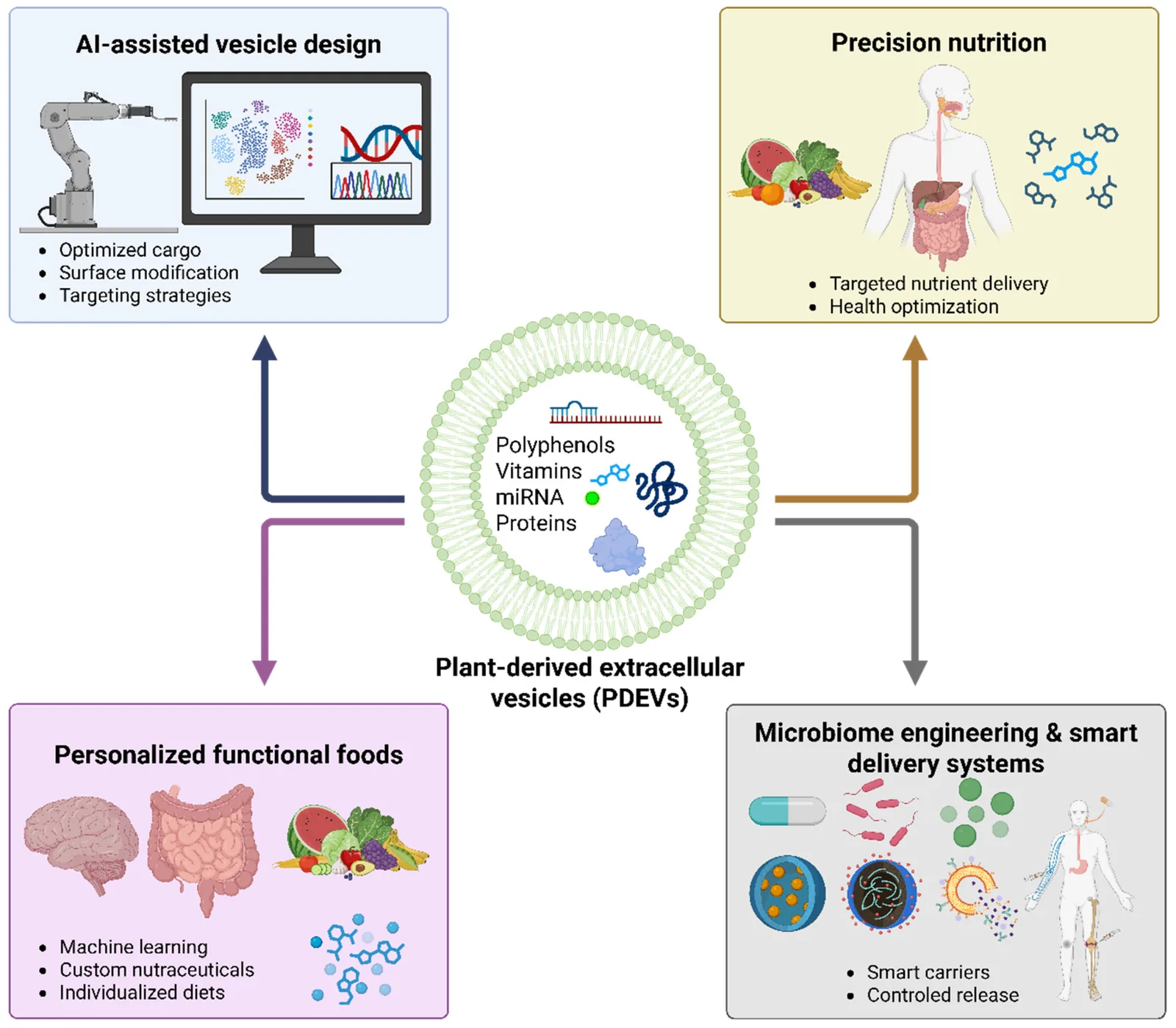
Emerging research directions for plant-derived extracellular vesicles (PDEVs). Colored panels represent AI-assisted design (blue), precision nutrition (yellow), personalized functional foods (purple), and microbiome engineering and smart delivery (gray). The central vesicle depicts molecular cargo; arrows indicate prospective applications, and icons illustrate relevant technologies and biological targets.Created in BioRender. Awlqadr, F. (2026) https://BioRender.com/7ntiymu.

Currently, the development of PDEVs is progressing rapidly, which will lead to changes in the methods for delivering functional foods and nutraceuticals. It is expected that there will be greater utilization of artificial intelligence (AI), precision nutrition, and microbiome-based methodologies to realize the full potential of these biological carriers. The approaches based on the use of artificial intelligence are being more widely adopted by researchers working on PDEV optimization, leveraging machine-learning techniques for the prediction of PDEV characteristics and behavior based on many omics datasets. An AI model also helps identify good plant sources and lipid compositions, thereby improving surface modification to make PDEVs stable and bioavailable. In this way, AI-assisted approaches may improve research efficiency and reduce unnecessary trial-and-error; however, their predictions require experimental validation before they can be used reliably for PDEV optimization or scale-up. Future AI-assisted research should move beyond prediction toward experimentally testable models that link plant source, processing conditions, vesicle composition, and biological performance. Comparative datasets generated under standardized isolation and characterization conditions could be used to determine whether AI models can reliably predict PDEV yield, cargo composition, loading efficiency, and cellular uptake. Experimental validation of these predictions would provide a measurable framework for assessing the usefulness of AI in PDEV development. Precision nutrition represents a potential application area in which PDEV composition and bioactive cargo could eventually be considered alongside individual metabolic, dietary, microbial, and physiological characteristics. However, PDEV-based precision nutrition remains an emerging concept rather than an established clinical or nutritional strategy. Future studies should determine whether differences in PDEV composition, dose, absorption, and biological exposure produce reproducible individual responses before personalized PDEV-based interventions can be considered. PDEV-based delivery mechanisms can be developed and implemented to address specific dietary deficiencies in individuals and to prevent the occurrence of certain diseases. PDEVs can achieve this by carrying lipids, proteins, and regulatory RNAs, which is relevant to the concept of advanced nutritional therapy. The same concept is relevant to the development of personalized functional food, where PDEVs are incorporated into customized meals tailored to individuals based on genetic background, microbial composition, and lifestyle factors to deliver nutraceuticals effectively and meet personalized requirements. One important area of research is to analyze whether changes in the composition of PDEV contribute to differential biological responses among organisms. Future experiments should focus on comparing PDEV preparations with various known molecular constituents in standardized models comprising intestinal, metabolic, and immune systems to determine whether specific cargo components can predict the type of nutritional or therapeutic response. The outcomes of this research could help determine whether PDEV composition and associated bioactive cargo contribute to measurable differences in nutritional or biological responses compared with conventional delivery approaches. Moreover, the development of functional significance of PDEVs together with microbial engineering provides new pathways for the modification of gut microbiome through the delivery of plant- origin miRNAs and other metabolites, thus influencing the host–microbiome interactions and improving health outcomes as a result of inter-kingdom communication, which represents another opportunity for improving inflammatory, metabolic, and immune homeostasis. Nonetheless, a significant but unresolved question is whether changes in the microbiome can be attributed to intact PDEVs or to their released cargo molecules, or to environmental changes in the intestines. In this compilation of studies, controlled studies that couple defined vesicle preparations with cargo-depleted or cargo-modified counterparts, along with standardized microbiome models, are necessary to address that issue. Another important finding in this line of study is that PDEVs can be combined with smart delivery systems, including stimuli-responsive hydrogels, nanoemulsions, and bioresponsive carriers. In this case, active substances can be released from PDEVs when environmental conditions, such as pH or the presence of enzymes, favor their release. In this way, hybrid systems become more effective at targeting, protecting sensitive cargo from environmental harm, and increasing the therapeutic efficacy of PDEVs. Key characteristics of PDEVs and conventional nano-delivery systems are compared in Table 9; these comparisons should be interpreted according to the intended application rather than as evidence of universal superiority. PDEVs possess naturally complex molecular structures and offer advantages in biological compatibility and food applicability. However, complexity complicates the meticulous control of composition, dosage, loading efficiency, and production. Meanwhile, liposomes and polymeric nanoparticles offer optimized control over composition, while nanoemulsions provide useful solubilization techniques. Thus, the fabrication of PDEVs must be undertaken with specific goals and applications in mind, rather than the thought that PDEVs are fundamentally more useful than conventional systems. Importantly, the edible nature of a source plant does not by itself establish the safety of purified, concentrated, drug-loaded, surface-modified, or parenterally administered PDEV preparations. Current evidence is dominated by in vitro and animal studies, and systematic human exposure and clinical evidence remain limited. Future safety assessment should therefore address dose- and route-specific toxicity, allergenicity, potential contaminants and co-isolated components, pharmacokinetics, tissue distribution and accumulation, immunological responses, and gastrointestinal fate. For translation, additional attention is required to batch-to-batch consistency, product-release specifications, scalable manufacturing, good manufacturing practice (GMP)-compatible production, and application-specific regulatory requirements. The implementation of PDEVs into practice should include provisions for stepwise development. The process should begin with the selection of standardized plant species, growing and processing conditions, and isolation techniques. Secondly, the constructs must be tested for specific physicochemical and molecular parameters, as well as for biological activity. Thirdly, the required studies should be conducted in accordance with accepted protocols to evaluate a variety of aspects important to PDEV’s application: reproducibility, stability, degradation in the gastrointestinal tract, dose-exposure response, and safety. Fourthly, a range of production techniques, such as tangential flow filtration, must be compared to determine whether it is possible to achieve the vessel’s yield, purity, content, and activity after moving to industrial production. Lastly, regulatory classification, quality management, product consistency, preparation-specific safety evaluation, and evidence of efficacy should be established before PDEV products can progress toward clinical or commercial applications. The key research objectives can thus be related to the following points: (i) deriving standardized methods for the extraction and characterization of the PDEV components; (ii) identifying the different cargo ingredients that can lead to the necessary biological effect; (iii) creating consistent and scalable manufacturing technologies; (iv) verifying the dosage, stability, and safety of the developed PDEV products; (v) assuring validity of the AI-predictions; and (vi) defining regulation parameters and quality standards for PDEV foods, nutraceuticals, and medicines. Addressing these research priorities would provide a structured pathway for evaluating whether laboratory findings can be translated into reproducible and practically applicable PDEV-based products Figure 11.

## 10. Conclusions

PDEVs are a new category of protein-, lipid-, RNA-, and phytochemical-based submicron carriers resulting from an evolutionary convergence between nutritional science and biomedicine, and they possess both structural integrity and inherent biological activity. PDEVs can act as therapeutic agents and carriers, providing a mechanism for targeted delivery and enhanced bioavailability of bioactive compounds, and supporting cross-kingdom communication to complement traditional dietary and therapeutic approaches. This article has discussed recent advances in understanding PDEV biogenesis, molecular composition, and functional diversity in edible and medicinal plants. Using emerging engineering techniques such as cargo loading, surface functionalization, and hybrid nanostructures, PDEVs have the potential for targeted delivery and personalized health applications. The ability of PDEVs to influence gut microbiota, promote nutrient uptake, and exhibit antioxidants, anti-inflammatory, and anti-cancer properties supports their potential use as foundational components of next-generation functional foods and precision nutrition systems. Despite these significant advances, numerous obstacles remain, including the standardization of isolation procedures, scalability for mass production, and comprehensive safety evaluation of PDEVs for widespread clinical and commercial use. These critical obstacles will need to be addressed if we are to realize the full translational potential of PDEVs. In summary, PDEVs represent a novel nanotechnology-based platform for protecting, delivering, and enhancing the biological availability of bioactive compounds, bridging naturally occurring bioactive substances with innovative biopharmaceutical delivery technologies and supporting the development of advanced, evidence-based health solutions for promoting health, disease prevention, and personalized nutrition.

## Figures and Tables

**Table 1:** Sources and comparative profiling of plant-derived extracellular vesicle and nanovesicle preparations from edible fruits and vegetables.

Plant Source	EV Type	Key Molecules	Size (nm)	Main Result	Reference
Grape	GELNs	Phenolics	~160 nm	Anti-inflammatory, low toxicity, stable	[33]
Grapefruit	GEVs	Proteins (HSP70 loading)	31.64–139.27 nm	Efficient drug delivery to glioma cells	[34]
Tomato	TEVs	Mixed biomolecules	14.65–54.71 nm	Larger vesicles but lower delivery efficiency	
Papaya	PDENs	Phenolics (DDMP)	~168 nm	Anti-inflammatory (↓ IL-6, ↑ IL-10)	[35]
Garlic	GENs	Lipids, proteins, miRNAs	NR	Improves colitis, modulates microbiota	[36]
Green onion	ELNs	Bioactive regulatory molecules	~167 nm	Inhibits ferroptosis & oxidative damage	[37]
Broccoli (*Brassica oleracea*)	(BDNs)	Sulforaphane-rich lipids	18.3–118.2 nm	Activated AMPK and prevented colitis	[31]
Lemon (*Citrus limon*)	Citrus-derived nanovesicles	Proteins, lipids, miRNAs	50–70 nm	Induced apoptosis in cancer cells	[38]
Strawberry (*Fragaria × ananassa*)	Exosome-like nanoparticles	Vitamin C, miRNAs, lipids	~100 nm	Reduced oxidative stress in stem cells	[39]
Orange (*Citrus sinensis*)	Orange EVs	Lipids, mRNA cargo	50–150	Delivered the mRNA vaccine effectively	[40]
Blueberry (*Vaccinium* spp.)	Blueberry EVs	Proteins, lipids, RNAs	50–220	Stable and modulated intestinal gene expression	[32]
*Physalis peruviana* (*P. peruviana*)	Exosome-like Nanoparticles (PDEN)	Collagen I, MMP-1, Proteins (170–1395 μg/mL)	190–220	PENC induces HDF proliferation and migration, upregulates collagen I production, and decreases MMP-1 levels.	[41]

↑, increase; ↓, decrease.

**Table 2:** Sources and comparative profiling of plant-derived extracellular vesicles (PDEVs) from medicinal plants.

Plant Source (Scientific Name)	EV Type	Key Molecules	Size (nm)	Main Result (Short)	Reference
*Zingiber officinale*	GELNs	DG (32:2), PG (23:2)	142–156	Lipids regulate B-cell uptake via protein binding.	[9]
*Panax ginseng*	GDEs	Ginsenosides (Rb1, Rg1, Rg3)	163–176	Reduces inflammation and protects against sepsis.	[44]
*Aloe arborescens*, *Zingiber officinale*, *Nigella sativa*	PDEVs	Drug-loaded vesicles	103–141	Enables BBB crossing and glioblastoma drug delivery.	[52]
*Zingiber officinale*	Modified GELNs	Sunitinib + intrinsic cargo	154–155	Inhibits RCC via targeting and immune modulation.	[53]
*Aloe arborescens*, *Aloe barbadensis*, *Aloe chinensis*	EVs	Anthraquinones	30–1000 (reported range)	Induces ROS-mediated melanoma phototoxicity.	[47]
*Zingiber officinale*	GELNs	miRNAs (aly-miR396a-5p)	NR	Suppresses SARS-CoV-2 lung inflammation.	[54]
*Zingiber officinale*	ZODENs	Lipids	200–400	Exhibits lipid-driven antibacterial activity.	[55]
*Curcuma amada*	MGDNVs	FSTL1-related cargo	231–507 (reported range)	Accelerates diabetic wound healing.	[56]

NR: not reported. Size values are presented as reported in the cited studies and are identified as a range or approximate mean where specified by the original study.

**Table 3:** Practical comparison of major PDEV isolation and purification strategies.

Method	Yield/Recovery	Purity	Processing Time	Scalability	Cost/Equipment	Vesicle Integrity	Refs.
Differential ultracentrifugation (DUC)	Moderate–high; protocol-dependent	Moderate	Long	Low–moderate	High equipment requirement	Moderate	[59,60,61,62,63]
Density-gradient ultracentrifugation (DGUC)	Moderate; possible losses during fractionation	High	Long	Low	High equipment requirement	Generally good	[60,64,65,66]
Size-exclusion chromatography (SEC)	Moderate–low	High	Moderate	Low–moderate	Moderate	High; relatively gentle	[67,68]
Ultrafiltration/TFF	High for concentration/recovery; protocol-dependent	Moderate	Short–moderate	High, particularly TFF	Moderate	Moderate; shear-dependent	[30,59,69]
Microfluidics	Protocol/device-dependent	Moderate–high; device-dependent	Short	Currently limited	High initial device requirement	Generally favorable under controlled conditions	[70,71]

**Table 4:** Functional food, nutraceutical, and therapeutic applications of plant-derived extracellular vesicles relevant to the delivery of bioactive compounds.

Type	Plant Source	EV Molecules/Cargo	Size (nm)	Delivery Type	Main Result	Reference
Targeted therapeutic platform	Ginger	Folic acid-modified GDEVs; native bioactives	241–268	Folate receptor-targeted delivery	Targeted M1 macrophages, promoted M2 polarization, and reduced RA symptoms	[102]
Colon-targeted siRNA system	Ginger	TNF-alpha siRNA and 6-shogaol	98–511	Oral EVs@ZIF-8@siRNA	Protected cargo in the stomach, lowered colonic TNF-alpha, and restored microbiota	[103]
Nutraceutical carrier	*Lentinula edodes*	Curcumin	<200	Oral vesicle delivery	Improved curcumin stability, gut retention, and microbiota-driven biotransformation	[104]
Colonic nutraceutical system	Banana juice	Curcumin	50–250	EV-loaded alginate/pectin beads	Delayed release and improved bioaccessibility, stress resistance, and UC potential	[105]
Anti-inflammatory carrier	Tomato fruit	Proteins and curcumin	146–180	Direct, sonication, or extrusion loading	Reduced IL-1beta expression; sonication enhanced anti-inflammatory activity	[106]
siRNA delivery platform	Grapefruit juice	siRNA	122	Microfluidic EV loading	Delivered siRNA intracellularly and achieved gene suppression	[100]
Oral mRNA vaccine	*Citrus sinensis* orange juice	SARS-CoV-2 S1 mRNA	50–150	Lyophilized oral capsules/gavage	Stable for one year and induced systemic and mucosal immune responses	[40]
Vaccine antigen carrier	Grapefruit/mandarin	GFP mRNA, AF-HSP70, HBsAg	46 ± 8.5; 227 ± 6.4	Oral HBsAg-loaded PENVs	Generated higher serum IgG than free HBsAg	[101]
Carotenoid delivery system	Broccoli	Astaxanthin	191.60 ± 2.23	BEV-coated PLGA nanoparticles	Improved formulation properties and anti-cancer activity	[107]

**Table 5:** Applications of plant-derived extracellular vesicles relevant to nutrients in diet.

Type	Plant Source	EV Molecules/Cargo	Size (nm)	Application/Disease	Delivery Type	Main Result	Reference
Functional food composition profiling	*Portulaca oleracea* L.	small-molecule metabolites, lipids, and proteins; terpenoids, citric acid, phosphatidylcholine, and ceramide	139 nm (D-PELNs); 186 nm (F-PELNs)	functional foods and compositional quality	Food-matrix-derived ELNs from dried and fresh plant material	Multi-omics analysis showed distinct metabolite, lipid, and protein profiles between dried and fresh Portulaca-derived ELNs.	[108]
Functional food	*Citrus sinensis* (Newhall sweet orange)	Native cargo	26.9–42.7 nm (preservation-dependent)	Antioxidant activity and nutraceutical preservation	In vitro exposure of human cells	Exhibited radical-scavenging activity and promoted fibroblast proliferation; preservation altered vesicle size and biological activity.	[111]
Dietary mineral-regulation application	tomato	proteins, nucleic acids, and miRNAs (sly-miR-168a, sly-miR-390a, and sly-miR-9476)	93 nm	intestinal zinc transport	Tomato EV uptake by differentiated intestinal epithelial cells	Tomato-derived EVs increased basolateral zinc transport while reducing MT-related mRNAs and intracellular zinc in differentiated epithelial cells.	[112]
Nutrient-rich beverage delivery system	navel, green, bingtang, and blood orange juices	Curcumin, resveratrol, shikonin, and apigenin; native lipid and protein components	A few hundred nanometers	full plant-based functional food and antioxidant beverage	Oral delivery in a polyphenol-enriched functional orange beverage	Orange EVs improved curcumin solubility, stability, and bioaccessibility; polyphenol-rich orange beverages exhibited greater antioxidant activity than blank beverages.	[113]
Functional food intestinal-protection application	*Ulva lactuca*	Not reported	188.8 ± 70.5 nm	high-fat diet-induced intestinal damage	UEV treatment as food-derived nanostructures	UEVs reduced ROS and apoptosis, restored tight junction proteins, and activated Nrf2/Keap1/HO-1 signaling under HFD-mimicking stress.	[114]
Functional food stability and gut uptake	blueberry	chemical compounds, protein, and total RNA	88.2 ± 7.7 nm	intestinal Caco-2 cell regulation and processing stability	Oral delivery through digestion-stable B-EVs	B-EVs remained stable during storage, heating, and digestion and were internalized by Caco-2 cells, affecting intestinal gene expression.	[32]
Hybrid antioxidant nutraceutical system	grapes and tomatoes	antioxidant compounds	Not reported	oxidative stress protection	Hybrid plant-derived nanovesicles	GT-HNVs improved stability, radical-scavenging activity, cellular uptake, and antioxidant protection against mitochondrial oxidative damage.	[115]
Nutrigenomic and antioxidant application	*Opuntia ficus-indica* fruit juice	bioactive phytocompounds	approximately 114 nm	antioxidant, anti-inflammatory, and nutrigenomic properties	FicoVes vesicle delivery	FicoVes reduced ROS, modulated inflammatory cytokines, and accelerated epithelial wound closure in vitro.	[116]
Functional food metabolic-support application	*Eruca sativa* leaves (arugula)	polyphenols and organic acids	Not reported in the uploaded record	metabolic syndrome-related oxidative stress and lipid/glucose metabolism	ALV supplementation	ALVs improved glucose tolerance and reduced total cholesterol, LDL, and hepatic injury biomarkers in HFHF-fed mice without toxicity.	[11]

**Table 6:** Applications of plant-derived extracellular vesicles relevant to probiotics and synbiotics delivery.

Type	Plant Source	EV Molecules/Cargo	Size (nm)	Application/Disease	Delivery Type	Main Result	Reference
Microbiota-manipulating ELNs	*Pueraria lobata*	Lipids; gma-miR4412	NR	Rheumatoid arthritis	Dietary ELNs	Suppressed *R. gnavus* PDC/phenylethylamine and arthritis	[120]
Symbiotic-supporting ELNs	Garlic	Proteins, miRNAs, peu-MIR2916-p3	79.6	Colitis; *B. thetaiotaomicron*	Oral	Promoted beneficial bacteria and reduced dysbiosis	[121]
SCFA-restoring supplement	Citrus	NR	NR	HFD dysbiosis	Oral CELNs	Reduced pathogens and increased beneficial bacteria/SCFAs	[122]
Microbiome-remodeling food	Tangerine peel	NR	NR	DSS intestinal inflammation	TPELN intervention	Reduced inflammation and improved microbiota/metabolites	[123]
Probiotic-fostering EVs	*Andrographis paniculata*	Hex1Cer, PG, PC, PI	50–400; mean 180	Colitis; *L. murinus*	Oral, 200 µL/day	Enriched *L. murinus* and preserved gut barrier	[124]
Colitis nanotherapeutic	Chinese bayberry	Lipids, proteins, miRNAs, polyphenols	100–300	DSS colitis	Oral, 1 × 10^9^ particles/kg/day	Restored barrier and enriched SCFA-producing bacteria	[125]

**Table 7:** Anti-cancer applications of native and engineered plant-derived extracellular vesicles (PDEVs).

Type	Plant Source	EV Molecules/Cargo	Size (nm)	Cancer Type	Delivery Type	Main Result (Shortened)	Reference
Plant EVs	Lemon (*Citrus limon*)	Proteomic cargo; TRAIL-related activity	50–70	CML and other tumors	In vitro/in vivo	Inhibited proliferation and CML growth via apoptosis	[38]
Engineered plant EVs	Lemon	DOX-loaded, heparin-cRGD EVs (HRED)	151.63	Drug-resistant ovarian cancer	Targeted engineered delivery	Enhanced uptake and reduced MDR through ATP depletion/efflux inhibition	[130]
Plant EV-like NPs	Ginseng (*Panax ginseng*)	Ceramides and immunomodulatory proteins	215.2	Melanoma	In vivo immunotherapy	Reprogrammed M2 to M1 macrophages and suppressed tumors	[131]
Plant ENs	Grape callus	trans-δ-Viniferin, miRNAs and proteins	90–250	Triple-negative breast cancer	In vitro	Reduced viability and induced G1 arrest and apoptosis	[132]
Plant ELNs	*Salvia chinensis* Benth	Genipin, EPA, lipids and phenylpropanoids	154.8–162.9	Hepatocellular carcinoma	Intratumoral, 1.5–3 mg/kg	Induced ferroptosis through AMPK/Nrf2/xCT and suppressed tumor traits	[133]
Plant EVs	*Platycodon grandiflorum*	Platycodin D, RNAs, proteins and lipids	73–74	Triple-negative breast cancer	Oral/IV, 5–10 mg/kg	Induced ROS/apoptosis, repolarized TAMs and improved immunity/microbiota	[134]
Plant ELNs	*Eutrema japonicum*	Ceramide, Hex1Cer, naringenin and genistein	200–210	Anti-cancer tumor model	Oral, 15 mg/kg × 5	Activated dendritic and CD8+ T cells and suppressed tumors	[109]
Engineered plant EVs	Black soybean	DOX-loaded Blex with cRGD	114.3	Breast cancer	IV, 2 × 10^9^ particles/3 days	Enhanced tumor uptake, cytotoxicity and in vivo tumor reduction	[135]

**Table 8:** Anti-inflammatory applications of plant-derived extracellular vesicles (PDEVs).

Type	Plant Source	EV Molecules	Size (nm)	Inflammation Type	Delivery Type	Main Result (Short)	Reference
EVs	*Prunus mume*	miRNA (miR159)	87–124 nm	Colitis (NLRP3)	Oral	Inhibits NLRP3 inflammasome, reduces IL-1β	[138]
EVs	*Coix lacryma-jobi*	RNAs, proteins	157 nm	Ulcerative colitis	Oral	↓ TNF-α, IL-6, ROS; ↑ IL-10, HO-1	[141]
EVs	*Beta vulgaris*	miRNA, proteins	82 nm	Inflammation/wound	Cellular uptake	Reduces inflammation, promotes healing	[144]
EVs	*Artemisia argyi*	miRNA (MIR2916)	139.7 nm	Liver inflammation	Oral	Inhibits TLR4/NLRP3, ↓ cytokines	[139]
EVs	*Coptis chinensis*	Polyphenols, miRNA	154.8 nm	Wound inflammation	Microneedle	M2 macrophage polarization, healing	[140]
EVs	*Cynara cardunculus*	Proteins, metabolites	NR	NAFLD inflammation	Cellular	↓ ROS, NO; activates SIRT1/AMPK	[137]
EVs	*Hordeum vulgare*	Antioxidant molecules	NR	LPS inflammation	In vitro	↓ NO, TNF-α, IL-1β	[142]
EVs	Polyphenol-loaded EVs	Polyphenols	145 nm	Neuroinflammation	Oral	Reduce oxidative stress (gut–brain axis)	[143]

↑, increase; ↓, decrease; NR, not reported.

**Table 9:** Comparison of plant-derived extracellular vesicles (PDEVs) with conventional nano-delivery systems data synthesized from [2,7,73,74].

Feature	PDEVs (Plant-Derived Extracellular Vesicles)	Liposomes	Nano Emulsions	Polymeric Nanoparticles
Origin	Natural (plant cells: fruits, vegetables, herbs)	Synthetic (phospholipids)	Synthetic/semi-natural (oil–water systems)	Synthetic (PLGA, chitosan, etc.)
Biocompatibility	Reported as favorable in several experimental models; preparation-, source-, dose-, and route-dependent	Generally well investigated; formulation- and application-dependent	Formulation- and surfactant-dependent	Variable (may induce toxicity)
Cargo Types	Lipids, proteins, miRNA, phytochemicals	Hydrophilic & lipophilic drugs	Mainly lipophilic compounds	Drugs, genes, proteins
Stability in the GI tract	Stability has been reported for selected preparations under simulated GI conditions; source-, processing-, and formulation-dependent	Variable depending on lipid composition and formulation	Low–moderate (unstable under digestion)	High (depending on polymer type)
Targeting ability	Potential for biological interactions and targeting; mechanisms remain under investigation	Limited (requires modification)	Poor (nonspecific distribution)	High (requires functionalization)
Bioavailability	Potentially enhanced; depends on cargo, formulation, and biological model	Moderate improvement	Improved solubility but limited absorption	High, but depends on the formulation
Loading efficiency	Endogenous cargo loading occurs naturally; additional loading strategies have been investigated, but quantitative capacity is preparation- and cargo-dependent	High on drugs	Moderate	High
Scalability	Challenging (standardization issues)	Well-established	Easy to scale	Scalable but costly
Toxicity	Requires preparation-, cargo-, dose-, and route-specific safety evaluation; available evidence is predominantly preclinical	Low–moderate	Surfactant-related toxicity possible	Polymer degradation toxicity possible
Regulatory status	Emerging (not fully standardized)	Approved in many applications	Widely used in food/pharma	Several FDA-approved systems
Functional food suitability	High potential for food applications; safety and processing requirements require further validation	Limited (synthetic perception)	Widely used in foods	Limited (more pharmaceutical)

## Data Availability

No new data were created or analyzed in this study. Data sharing is not applicable to this article.

## Supplementary Materials

The following supporting information can be downloaded at: https://www.mdpi.com/article/10.3390/molecules31203754/s1, Table S1: Complete database-specific search strategies.
