# Supplementary material for: Next-Generation Functional Nanoplatforms: Engineering Plant-Derived Extracellular Vesicles for Targeted Bioactive Delivery and Health Applications
Source: Molecules. 2026 Oct 19;31(20):3754. doi: 10.3390/molecules31203754 (PMC13707648; doi:10.3390/molecules31203754)
Supplement: Supplementary file 1 [file molecules-31-03754-s001.zip › molecules-4547474-supplementary.pdf]

# Supplementary Search Strategies for Plant Derived Extracellular Vesicles

*Supplementary Table S1*

**Search coverage.**

| Database | Platform | Last searched | Records |
|---|---|---|---|
| PubMed | NLM | 1 Apr 2026 | 745 |
| Scopus | Elsevier | 1 Apr 2026 | 3,498 |
| Embase | Embase.com | 1 Apr 2026 | 858 |
| Web of Science Core Collection | Clarivate | 1 Apr 2026 | 1,610 |

**PRISMA totals.** Records identified: 6,711; duplicates removed: 3,198; records screened: 3,513; reports assessed: 270; studies included: 142.

## PubMed

*Fields: MeSH and Title/Abstract.*

```
(
 (
  "Extracellular Vesicles"[Mesh]
  OR exosome*[Title/Abstract]
  OR "extracellular vesicle"[Title/Abstract]
  OR "extracellular vesicles"[Title/Abstract]
  OR "extracellular nanovesicle"[Title/Abstract]
  OR "extracellular nanovesicles"[Title/Abstract]
  OR "exosome-like nanoparticle"[Title/Abstract]
  OR "exosome-like nanoparticles"[Title/Abstract]
  OR "exosome-like nanovesicle"[Title/Abstract]
  OR "exosome-like nanovesicles"[Title/Abstract]
  OR "vesicle-like nanoparticle"[Title/Abstract]
  OR "vesicle-like nanoparticles"[Title/Abstract]
  OR nanovesicle*[Title/Abstract]
 )
 AND
 (
  plant*[Title/Abstract]
  OR "medicinal plant"[Title/Abstract]
  OR "medicinal plants"[Title/Abstract]
  OR "edible plant"[Title/Abstract]
  OR "edible plants"[Title/Abstract]
  OR fruit*[Title/Abstract]
  OR vegetable*[Title/Abstract]
  OR herb*[Title/Abstract]
  OR botanical*[Title/Abstract]
  OR crop*[Title/Abstract]
  OR "food-derived"[Title/Abstract]
  OR "food derived"[Title/Abstract]
 )
)
OR PDEV*[Title/Abstract]
OR PDEN*[Title/Abstract]
OR PELN*[Title/Abstract]
OR "plant-derived extracellular vesicle"[Title/Abstract]
OR "plant-derived extracellular vesicles"[Title/Abstract]
```

```
OR "plant-derived exosome"[Title/Abstract]
OR "plant-derived exosomes"[Title/Abstract]
OR "plant-derived exosome-like nanoparticle"[Title/Abstract]
OR "plant-derived exosome-like nanoparticles"[Title/Abstract]
OR "plant-derived nanovesicle"[Title/Abstract]
OR "plant-derived nanovesicles"[Title/Abstract]
OR "edible plant-derived nanoparticle"[Title/Abstract]
OR "edible plant-derived nanoparticles"[Title/Abstract]
```

## Scopus

*Field: TITLE-ABS-KEY; proximity: W/3.*

```
TITLE-ABS-KEY(
  PDEV* OR PDEN* OR PELN*
  OR "plant-derived extracellular vesicle*"
  OR "plant derived extracellular vesicle*"
  OR "plant extracellular vesicle*"
  OR "plant-derived exosome*"
  OR "plant derived exosome*"
  OR "plant exosome*"
  OR "plant-derived exosome-like nanoparticle*"
  OR "plant derived exosome-like nanoparticle*"
  OR "plant-derived nanovesicle*"
  OR "plant derived nanovesicle*"
  OR "plant-derived vesicle-like nanoparticle*"
  OR "edible plant-derived nanoparticle*"
  OR "edible plant-derived nanovesicle*"
  OR (
    (plant* OR "medicinal plant*" OR "edible plant*" OR fruit* OR vegetable*
     OR herb* OR botanical* OR crop* OR "food derived" OR food-derived)
    W/3
    (exosome* OR "extracellular vesicle*" OR nanovesicle*
     OR "exosome-like nanoparticle*" OR "vesicle-like nanoparticle*")
  )
)
```

## Embase

*Fields: Emtree /exp and title, abstract, keyword (:ti,ab,kw).*

```
(
  ('extracellular vesicle'/exp OR 'exosome'/exp
   OR exosome*:ti,ab,kw
   OR 'extracellular vesicle*':ti,ab,kw
   OR 'extracellular nanovesicle*':ti,ab,kw
   OR 'exosome-like nanoparticle*':ti,ab,kw
   OR 'exosome-like nanovesicle*':ti,ab,kw
   OR 'vesicle-like nanoparticle*':ti,ab,kw
   OR nanovesicle*:ti,ab,kw)
  AND
  ('plant'/exp OR 'fruit'/exp OR 'vegetable'/exp OR 'medicinal plant'/exp
   OR plant*:ti,ab,kw OR 'medicinal plant*':ti,ab,kw
   OR 'edible plant*':ti,ab,kw OR fruit*:ti,ab,kw
   OR vegetable*:ti,ab,kw OR herb*:ti,ab,kw OR botanical*:ti,ab,kw
   OR crop*:ti,ab,kw OR 'food-derived':ti,ab,kw OR 'food derived':ti,ab,kw)
)
OR PDEV*:ti,ab,kw OR PDEN*:ti,ab,kw OR PELN*:ti,ab,kw
OR 'plant-derived extracellular vesicle*':ti,ab,kw
OR 'plant derived extracellular vesicle*':ti,ab,kw
OR 'plant extracellular vesicle*':ti,ab,kw
OR 'plant-derived exosome*':ti,ab,kw
OR 'plant derived exosome*':ti,ab,kw
OR 'plant exosome*':ti,ab,kw
OR 'plant-derived exosome-like nanoparticle*':ti,ab,kw
OR 'plant derived exosome-like nanoparticle*':ti,ab,kw
OR 'plant-derived nanovesicle*':ti,ab,kw
OR 'plant derived nanovesicle*':ti,ab,kw
```

```
OR 'edible plant-derived nanoparticle*':ti,ab,kw
```

## Web of Science Core Collection

*Field: Topic (TS); proximity: NEAR/3.*

```
TS=(
 PDEV* OR PDEN* OR PELN*
 OR "plant-derived extracellular vesicle*"
 OR "plant derived extracellular vesicle*"
 OR "plant extracellular vesicle*"
 OR "plant-derived exosome*"
 OR "plant derived exosome*"
 OR "plant exosome*"
 OR "plant-derived exosome-like nanoparticle*"
 OR "plant derived exosome-like nanoparticle*"
 OR "plant-derived nanovesicle*"
 OR "plant derived nanovesicle*"
 OR "plant-derived vesicle-like nanoparticle*"
 OR "edible plant-derived nanoparticle*"
 OR "edible plant-derived nanovesicle*"
 OR (
  (plant* OR "medicinal plant*" OR "edible plant*" OR fruit* OR vegetable*
   OR herb* OR botanical* OR crop* OR "food derived" OR food-derived)
  NEAR/3
  (exosome* OR "extracellular vesicle*" OR nanovesicle*
   OR "exosome-like nanoparticle*" OR "vesicle-like nanoparticle*")
 )
)
```

## Deduplication

Records were merged and deduplicated using DOI, database accession number, PMID, and normalized title; uncertain matches were checked against author, year, journal, volume, and pages.
